# *βν* Integrin Inhibits Chronic and High Level Activation of JNK to Repress Senescence Phenotypes in *Drosophila* Adult Midgut

**DOI:** 10.1371/journal.pone.0089387

**Published:** 2014-02-20

**Authors:** Takashi Okumura, Koji Takeda, Kiichiro Taniguchi, Takashi Adachi-Yamada

**Affiliations:** 1 Department of Life Science, Faculty of Science, Gakushuin University, Tokyo, Japan; 2 Graduate Course in Life Science, Graduate School of Science, Gakushuin University, Tokyo, Japan; 3 Institute for Biomolecular Science, Gakushuin University, Tokyo, Japan; Seoul National University, Republic of Korea

## Abstract

Proper control of adult stem cells including their proliferation and differentiation is crucial in maintaining homeostasis of well-organized tissues/organs throughout an organism's life. The *Drosophila* adult midgut has intestinal stem cells (ISCs), which have been exploited as a simple model system to investigate mechanisms controlling adult tissue homeostasis. Here, we found that a viable mutant of *βν integrin* (*βint-ν*), encoding one of two *Drosophila* integrin β subunits, showed a short midgut and abnormal multilayered epithelia accompanied by an increase in ISC proliferation and misdifferentiation defects. The increase in ISC proliferation and misdifferentiation was due to frequent ISC duplication expanding a pool of ISCs, which was caused by depression of the Notch signalling, and up-regulation of *unpaired* (*upd*), a gene encoding an extracellular ligand in the JAK/STAT signalling pathway. In addition, we observed that abnormally high accumulation of filamentous actin (F-actin) was caused in the *βint-ν* mutant enterocytes. Furthermore, the defects were rescued by suppressing c-Jun N-terminal kinase (JNK) signalling, which was up-regulated in a manner correlated with the defect levels in the above-mentioned *βint-ν* mutant phenotype. These symptoms observed in young *βint-ν* mutant midgut were very similar to those in the aged midgut in wild type. Our results suggested that *βint-ν* has a novel function for the *Drosophila* adult midgut homeostasis under normal conditions and provided a new insight into possible age-related diseases caused by latent abnormality of an integrin function.

## Introduction

In maintaining homeostasis of well-organized adult tissues/organs, it is crucial to control proliferation and differentiation in adult stem cells, since adult stem cells constantly replenish new healthy differentiated cells constituting tissues/organs throughout an organism's life [1], [2]. Adult stem cells also have an essential capability that possibly alters their proliferation and differentiation rate in adapting to various environmental changes. Moreover, disruption of their control caused by internal or external factors including mutations, chemicals, and aging leads to or affects physiological dysfunction of tissues/organs and several diseases such as cancer [3]. Thus, it is important to understand the mechanism that preserves proper control of adult stem cells.

Intestinal stem cells (ISCs) in the small intestine of mammals have been well studied [4], [5]. The ISCs are located at the bottom of the crypts (a niche of ISCs), rapidly undergo self-renewal and provide some types of differentiated cells that constitute a monolayer epithelium and are finally excluded from the top of the villi in a turnover of 2–3 days. Similarly, the fruit fly *Drosophila* also has a population of ISCs scattered in the adult midgut, a counterpart of the small intestine [6], [7]. The *Drosophila* ISCs produce enteroblasts (EBs) that directly differentiate into two types of mature differentiated cells, i.e. enterocytes (ECs) and enteroendocrine (ee) cells, without further cell division in weekly turnovers [6], [7]. Both in mammals and *Drosophila*, ISC proliferation and differentiation are regulated by similar signalling pathways/factors including Wnt/wingless, Notch (N), epidermal growth factor (EGF), cytokines/JAK/STAT, c-Jun N-terminal kinase (JNK), Hippo and insulin in normal and regenerative conditions [8]–[19]. Thus, the *Drosophila* adult midgut should further provide understanding of the genetic mechanism regulating adult tissue homeostasis.

Integrins form heterodimers composed of α and β subunits and their extracellular domains are involved in cell adhesion to the extracellular matrix (ECM) as well as signal reception from the ECM [20]. The intracellular regions also interact with the actin cytoskeleton and several growth factor receptor signalling factor complexes that are involved in various cell events including proliferation, migration, apoptosis, and differentiation both in normal and disease conditions [21]. Furthermore, integrins play important roles in epithelial homeostasis since their impaired activities are related to several human epithelial disorders such as cancer, psoriasis, and epidermolysis bullosa pathologies [22]. However, the complex role of integrins in adult tissues/organs is not yet fully understood.

While the human genome has eight genes for integrin *β* subunits, the *Drosophila* genome encodes only two genes for these, *myospheroid* (*mys*) and *βν integrin* (*βint-ν*) [23], [24]. The former has been well studied and is probably ubiquitously expressed and involved in many developmental events including retention of stem cell population in testis and ovary [25], [26]. Most recently, *mys* was also reported to be involved in asymmetric division of ISCs [27] and required for maintenance and proliferation of ISCs [28]. On the other hand, a few studies about *βint-ν* reported that it was involved in phagocytosis of apoptotic cells and endoderm migration [29], [30]. With regard to the ISC functions recently reported, *βint-ν* only plays a supplementary role when *mys* is disrupted [28].

Here, we found that the young adult midgut of a *βint-ν* mutant showed a shrinking and multilayered epithelium accompanied by expansion of the ISC pool and misdifferentiation of epithelial cells. The defects are locally induced at first and rapidly spread through the midgut with age by up-regulation of *upd* and JNK signalling that promote turnover of the epithelium. This condition is very similar to that found in the aged midgut. Normal *βint-ν* collectively inhibits chronic high level activation of the JNK-Upds pathway to maintain homeostasis of the *Drosophila* adult midgut.

## Results

### *βint-ν* expression in all types of the adult midgut epithelial cells

Although homozygous null mutants of *βint-ν* have been reported to be viable and fertile, *βint-ν* was found to be evolutionally conserved among *Drosophila* species (Figure S1). In addition, *βint-ν* is exclusively expressed in the embryonic and larval midgut including imaginal island, a progenitor of the adult midgut epithelia [24]. More recently, *βint-ν* mRNA was also detected from the adult midgut with RT-PCR (Lin et al., 2013). Therefore, we speculated an additional novel function of *βint-ν* responsible for keeping the adult midgut healthy. To examine this possibility, we performed the following experiments using female midguts in this study.

**Figure 1:**
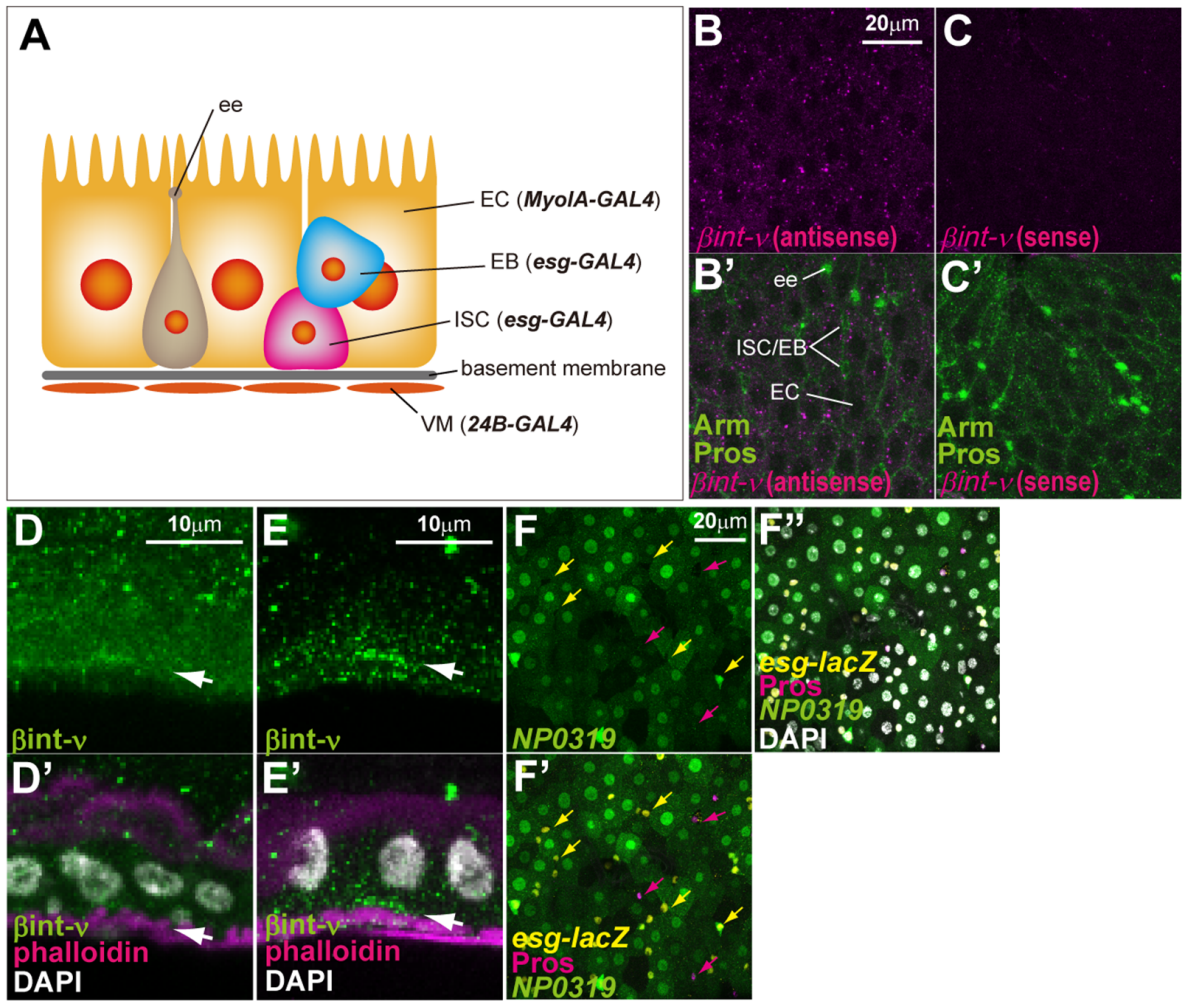
*βint-ν* is expressed in the adult midgut epithelium. (A) The diagram of the adult midgut composed of ISCs, EBs, ECs, ees, and VMs. The cell-type specific GAL4 drivers, *esg-GAL4*, *MyoIA-GAL4*, and *24B-GAL4*, used in this study are shown in parentheses. (B–C) Transcripts of *βint-ν* were detected with an antisense probe (magenta in B and B’) for *βint-ν* mRNA but not with a sense probe (magenta in C and C’) for it. “Pros” and “Arm” respectively indicate the ee nuclei and the outline of cells (green in B’ and C’). Small cells without Pros are ISC/EB. (D–E) βint-ν protein was detected with anti-βint-ν antibodies (green, D [30], E [24]). Basal visceral muscles were strongly marked with phalloidin staining (magenta in D’ and E’). Arrows indicate the distribution of βint-ν protein at the basal side. (F-F”) Expression of *NP0319* (a *βint-ν*-GAL4, green), monitored with *UAS-GFP*, was detected in polyploid ECs and a subset of *esg-lacZ*-positive cells (yellow) but not the other *esg-lacZ-* and Pros-positive cells (magenta). Yellow and magenta arrows indicate examples of *esg-lacZ-* and Pros-positive cells. Nuclei were stained with DAPI (white in D’, E’, and F”).

The adult midgut was composed of epithelial cells (ISCs, EBs, ECs, ees) and visceral muscles (VMs) (Figure 1A). First, we confirmed expression of *βint-ν* in the adult midgut with *in situ* hybridization experiment.The *βint-ν* mRNA was detectable in all types of epithelial cells including diploid cells (ISCs, EBs and ees) and polyploid cells (ECs) with antisense probe (Figure 1B) but not with sense probe (Figure 1C). In addition, *βint-ν* mRNA disappeared in the adult midgut epithelium by depletion of *GATAe* that activates expression of *βint-ν* [31] (to be presented elsewhere). Furthermore, distribution of βint-ν protein at the basal side of the epithelium was detected with immunostaining using two kinds of anti-βint-ν antibodies that had independently been generated (Figure 1D and 1E) [24], [30]. A fewer βint-ν protein was detected also at the visceral muscle (Figure 1D and 1E). Moreover, we also found that *NP0319*, a GAL4-enhancer trap construct inserted near the *βint-ν* locus, showed a strong expression of GFP driven by GAL4 specifically in ECs (Figure 1F). Collectively, these indicated that *βint-ν* was expressed in all types of the adult midgut epithelium.

### *βint-ν* mutants showed shortened and multilayered epithelium of adult midgut

**Figure 2:**
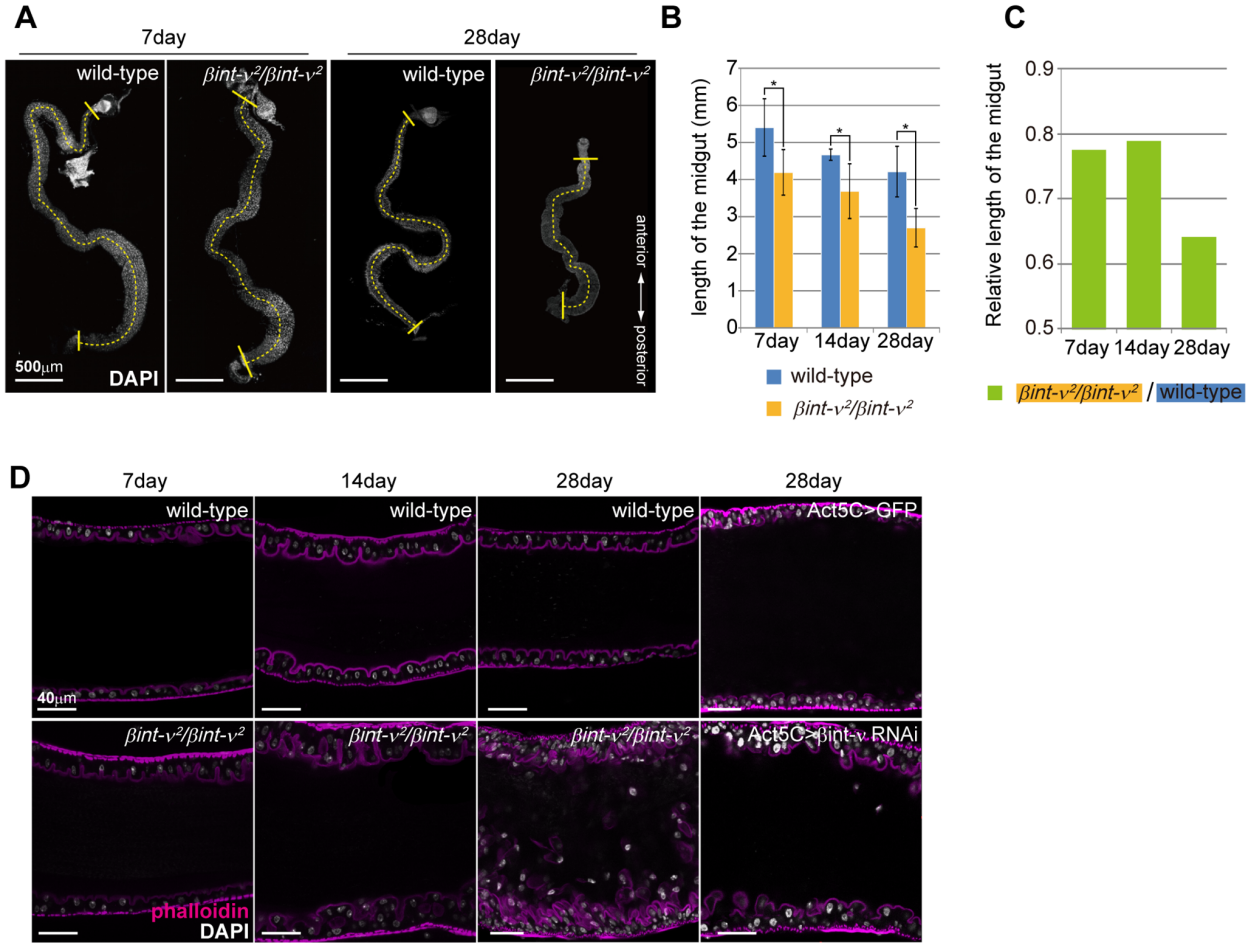
Adult midgut of *βint-ν* mutants showed shortened and multilayered epithelium. (A) The whole adult midgut of wild-type flies and *βint-ν^2^* homozygotes, stained with DAPI, at 7 and 28 days old. Broken lines were used to measure the midgut lengths. (B) The average of adult midgut length of wild-type flies and *βint-ν^2^* homozygotes. The error bars are standard error of means (S.E.M.). P-values were calculated by Student's t test, *p<0.05. (C) The *βint-ν^2^* adult midgut length relative to that of wild-type flies. (D) A cross-section view of the adult midgut of wild-type, *βint-ν^2^*, and control (*Act5C>GFP*) flies, and a knockdown of *βint-ν* (*Act5C>βint-ν RNAi*) flies, stained with Rhodamine-phalloidin (magenta) and DAPI (white).

To examine whether *βint-ν* has a function in maintenance of normal adult midgut, we observed the adult midgut morphology of homozygote flies for *βint-ν* ^2^, a null allele of *βint-ν* [29], and found that it was shorter than that of wild-type flies (Figure 2A). Both in wild-type and *βint-ν* ^2^ flies, the length gradually decreased with age, which was measured at 7, 14, and 28 days old (Figure 2B).Furthermore, the decrease rate was higher in *βint-ν* ^2^ flies from 14 to 28 days old (Figure 2C).

Next, we focused on the posterior region of the midgut (PMG) of wild-type and *βint-ν* ^2^ flies at 7, 14, and 28 days old, because substantial shortening was observed in this region (Figure 2A–C). In 7, 14, and 28-day-old wild-type flies, the epithelium constantly showed a monolayer feature (Figure 2D). In 7-day-old *βint-ν* ^2^ flies, the epithelium was also monolayer, but mild and severe abnormal multilayers were observed at 14 and 28 days old, respectively (Figure 2D). When a double strand RNA construct against *βint-ν* (*βint-ν* RNAi) was expressed with the *Act5C*-GAL4 driver, similar defects were induced in 28-day-old flies (Figure 2D). A time period of the multilayered defect was consistent with the number of days when severe shortening of the midgut observed. Thus a multilayered epithelium caused by depletion of *βint-ν* might be responsible for the shortening of the adult midgut. Also, above results suggested that *βint-ν* is required for maintaining morphological homeostasis of the adult midgut.

### *βint-ν* in ECs restricts proliferation of ISCs

**Figure 3:**
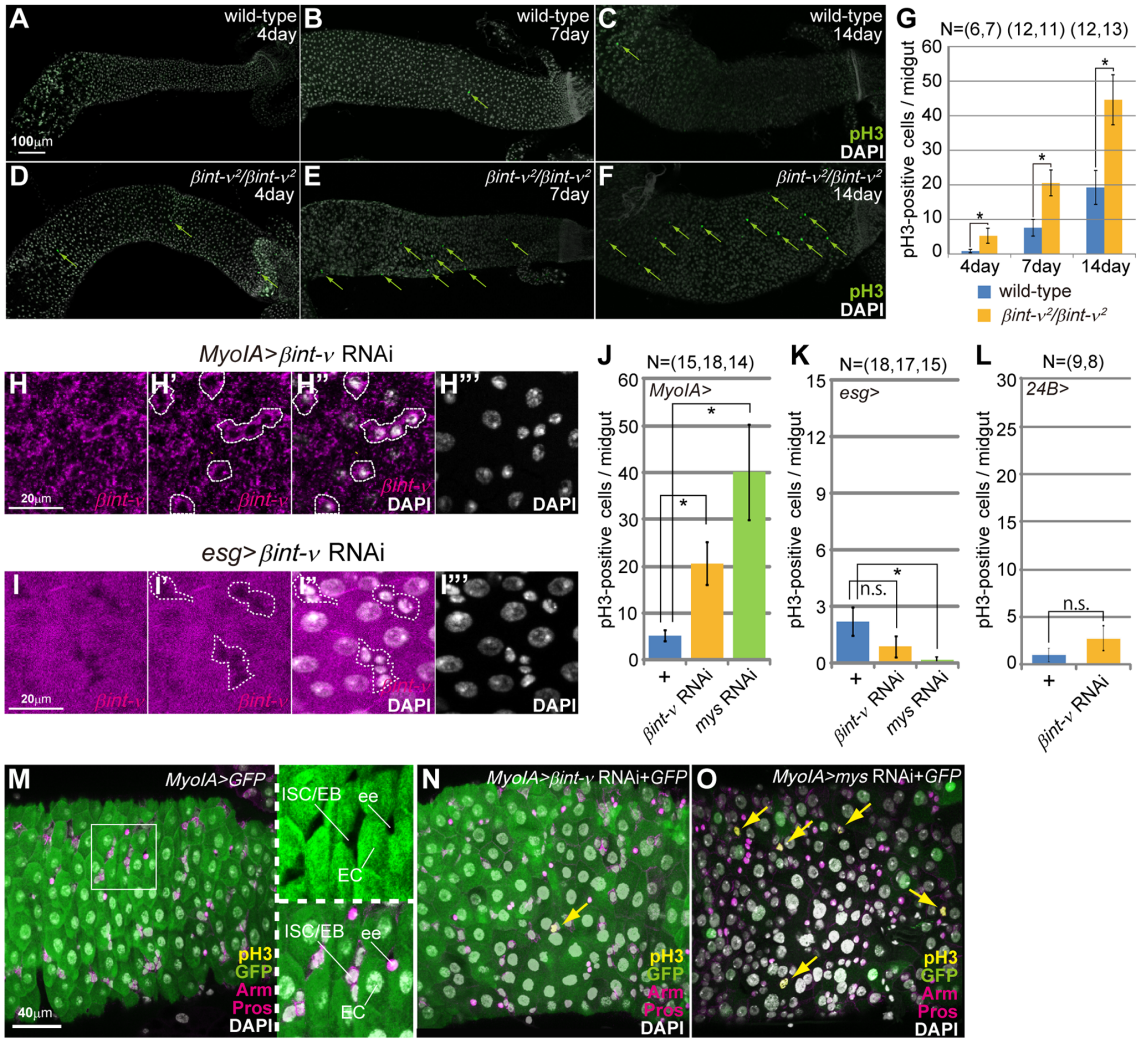
A function of *βint-ν* in ECs was involved in ISC proliferation. (A–B) The PMG of wild-type (A–C) and *βint-ν^2^* homozygote (D–F) flies at 4 (A and D), 7 (B and E), and 14 days old (C and F) stained with anti-pH3 antibody (green) and DAPI (white). Arrows indicate pH3-positive cells. (G) The average number of pH3-positive cells in the whole midgut of wild-type flies and *βint-ν^2^* homozygotes at 4, 7, and 14 days old. (H and I) The *βint-ν* mRNA (magenta) of the PMG epithelium treated with *βint-ν* RNAi using *MyoIA-GAL4* (H-H’’’) and *esg-GAL4* (I-I’’’). H and I are hybridization signals (magenta). H’’’ and I’’’ are nuclear staining by DAPI (white). Most of the cells with small nuclei, which are outlined with broken lines (H’, H’’, I’ and I’’), are considered to be ISCs/EBs. The outside of the outlined area is the cytoplasm of EC. The EC-specific and ISC/EB-specific reduction in *βint-ν* mRNA can be seen in H and I, respectively. (J–L) The average number of pH3-positive cells in the whole midgut treated either with non-RNAi (+), *βint-ν* RNAi, and *mys* RNAi for 7 days with *MyoIA-GAL4* (J), *esg-GAL4* (K), and *24B-GAL4* (L), respectively. (M–O) The PMG with non-RNAi (M), *βint-ν* RNAi (N), and *mys* RNAi (O) treatment using *MyoIA-GAL4* driver for 7days, stained with anti-pH3 (yellow), anti-GFP (green), anti-Arm, and anti-Pros (magenta) antibodies. (M) As previously reported, *MyoIA-GAL4* drove expression of UAS-target (GFP in this Figure) specifically in ECs but neither in ISCs/EBs (marked by Arm localization to cell membrane), nor in ees, (Pros-positive). Right two panels in M are magnifications of boxed area in the left panel. (N and O) Compared with *βint-ν* RNAi (N), *mys* RNAi (O) caused more increase of mitotic ISCs detected with anti-pH3 (yellow) antibody. Nuclei were stained with DAPI (white). In G and J–L, the error bars are S.E.M. and P-values were calculated by Student’s t test, *p<0.05.

Some studies have reported that the pathological multilayering of the epithelium was accompanied by an increase in mitotic ISCs in the adult midgut [32]–[34]. Therefore, we speculated that ISC proliferation was also increased in *βint-ν* ^2^ adult midguts. To confirm this possibility, we counted the number of mitotic cells possessing phosphorylated histone H3 (pH3), a marker of M-phase chromosomes, in the whole midgut (Figure 3A–F). In wild-type flies, the average number of pH3-positive cells per midgut was 0.83±0.48, 7.58±2.42, and 19.25±4.92 at 4, 7, and 14 days old, respectively (Figure 3G). For *βint-ν* ^2^ homozygotes, the numbers were higher: 5.29±2.18, 20.55±3.72, and 44.62±7.24 at 4, 7, and 14 days old, respectively (Figure 3G). These averages were statistically different between wild-type and *βint-ν* ^2^ homozygote flies at each age. These results indicated that the normal gene product of *βint-ν* restricted the number of proliferating ISCs.

The adult midgut was composed of ISCs, EBs, ECs, ees, and VMs (Figure 1A). Next, to confirm which cell types required a function of *βint-ν* in restricting the number of mitotic ISCs, we performed cell type-specific *βint-ν* RNAi with a stage-specific forced expression system TARGET [35]. For this analysis, we used the following GAL4 drivers: *esg-GAL4* (ISC and EB), *MyoIA-GAL4* (EC), and *24B-GAL4* (VM), combined with *tub-GAL80^ts^*, a temperature-sensitive GAL4-repressor GAL80 (Figure 1A) [35]. The *βint-ν* RNAi induced by *esg-* and *MyoIA-GAL4* caused a decrease of the *βint-ν* mRNA specifically in ECs and ISCs/EBs, respectively (Figure 3H, and 3I). When *esg-GAL4* or *24B-GAL4* was used, we did not detect any statistically significant difference in the number of pH3-positive cells between the control and *βint-ν* RNAi-treated midguts (Figure 3K and 3L). By contrast, using *MyoIA-GAL4* resulted in a statistically significant increase in the number of mitotic ISCs. (Figure 3J). In this condition, any disruption of the basement membrane (BM) marked by Vkg-GFP, a GFP fusion of collagen IV, cannot be detected (Figure S2) [8]. From these results, we concluded that a function of *βint-ν* in ECs was involved in restricting the number of mitotic ISCs.

### *βint-ν* genetically interacts with Dl/N signalling factors in regulating ISC pool

**Figure 4:**
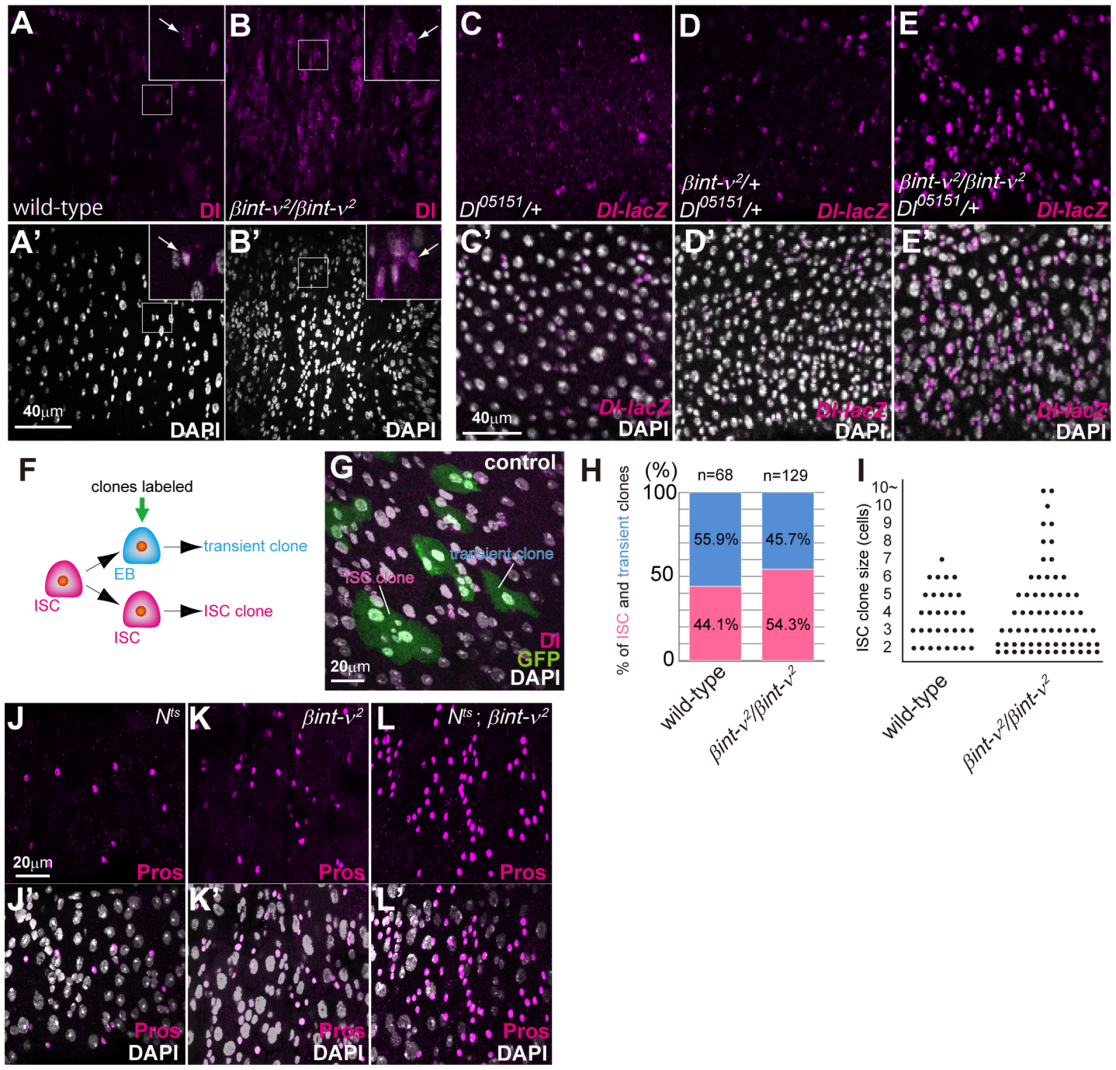
Frequent ISC duplication was caused in *βint-ν* mutant. (A–B) The PMG of 14-day-old wild-type flies (A and A’) and *βint-ν^2^* homozygotes (B and B’) stained with anti-Dl antibody (magenta in A, B, and insets). Dl was prominently observed in punctuates in wild-type flies (arrows in insets of A and A’). The diploid cells of *βint-ν^2^* homozygote predominantly showed Dl localization to the plasma membrane (arrows in insets of B and B’). (C–E) The PMG of seven-day-old *Dl^05151^*/+ (C and C’), *βint-ν^2^*/+, *Dl^05151^*/+ (D and D’), and *βint-ν^2^*/*βint-ν^2^*, *Dl^05151^*/+ (E and E’) flies stained with anti-β-gal antibody (magenta in C–E) and DAPI (white in C’, D’, and E’). (F) Either transient or ISC clones were labelled by the MARCM system. (G) An example of the PMG showing transient and ISC clones labelled by GFP (green). The ISC clones but not the transient clones contained Dl-positive ISC (magenta). (H) The ratio of transient (blue bar) and ISC (magenta bar) clones generated in control (*FRT19A, tub-GAL80, hs-FLP/FRT19A; Act5C-GAL4, UAS-GFP^S65T^/+*) or *βint-ν* ^2^ *(FRT19A, tub-GAL80, hs-FLP/FRT19A; βint-ν^2^/βint-ν^2^; Act5C-GAL4, UAS-GFP^S65T^/+*) midguts. (I) The ISC clone size in control and *βint-ν^2^* midguts at 5 days after clone induction. Each dot represents one clone. (J-L) The PMG of *N^ts^* homozygotes (J), *βint-ν^2^* homozygotes (K), and *N^ts^* and *βint-ν^2^* double homozygotes (L) stained with anti-Pros antibody (magenta). The ees marked by “Pros” increased more in the *N^ts^* – *βint-ν^2^* double mutant than in the *N^ts^* or *βint-ν^2^* single mutants. Nuclei were stained with DAPI (white in A’-E’, G, and J’-L’).

One possible reason for the increase in the number of mitotic ISCs was expansion of the ISC pool. To confirm this hypothesis, we first observed ISCs specifically marked by an anti-Delta (Dl) antibody in *βint-ν* ^2^ flies. Compared with wild-type, Dl-positive diploid cells increased in *βint-ν* ^2^ homozygotes (Figure 4A and 4B). Furthermore, these ISCs were frequently juxtaposed (Figure 4B and 4B’ insets), which is unusual in wild-type (Figure 4A and 4A’ insets), suggesting the occurrence of symmetric division of ISC. In addition, localization of Dl to the plasma membrane rather than to the cytoplasm was predominantly observed in *βint-ν* ^2^ flies, which was similar to that in the ISC-like tumour in the *neuralized* mutant where symmetric division occurred exclusively by inactivating Dl/N signalling (Figure 4B and 4B’ arrows in insets compared with 4A and 4A’ arrows in insets) [16]. Next, to recognize ISCs more clearly in the overcrowded epithelial cells of the *βint-ν* ^2^ midgut, we also used the *Dl-lacZ* strain that expresses β-galactosidase localizing to the nuclei. Consequently, we observed an increase in the ISC number from early stage (7 days old) (Figure 4E and 4E’ compared with 4C and 4C’). Similarly, pairs of ISCs were frequently observed in *βint-ν^2^* homozygotes (Figure 4E and 4E’). In addition, a lower angle between the spindle body and BM, which has been speculated to indicate symmetric division of the ISCs, was observed more often in *βint-ν* ^2^ flies than in wild-type flies (Figure S3) [36]. These observations suggested that the frequent duplication of the ISCs caused an expansion of the ISC pool in *βint-ν* mutant.

To further confirm whether frequency of ISC self-renewal was increased in *βint-ν* mutants, we generated a mosaic analysis with a repressible cell marker (MARCM, [37]) clone in control and *βint-ν* mutant midguts. In this analysis, the committed EB or ISC respectively produced a visible transient clone (composed of a single cell) or an ISC clone (composed of multiple cells) (Figure 4F and 4G). As expected, the frequency of ISC clone generation in *βint-ν^2^* homozygotes was higher than that in control flies (Figure 4H), indicating that the ratio of ISC self-renewal to total ISC division increased. A slight promotion of ISC clone size was consistently observed in *βint-ν^2^* mutants (Figure 4I). The ISC pool expansion was also consistent with the midgut shortening and multilayered epithelia, since *N* RNAi or *Su(H)* mutation caused a similar ISC increase and morphological defects [10], [32].

Recently, lateral inhibition by Dl/N was found to be involved in balancing ISC self-renewal and EB commitment rates, which likely affected the ISC population size [38]. We thus tested a genetic interaction between *βint-ν^2^* and *Dl^05151^* (the above-mentioned *Dl-lacZ* strain, also known as a loss of function allele) and confirmed a further increase in the ISC population (Figure 4C–E), suggesting a possible relationship between *βint-ν* and *Dl*. If N signalling decreased in *βint-ν* mutants, the ee population was also expected to expand, since strong activation of N signalling in EBs is known to inhibit ee differentiation [16]. Although the homozygotes of *N^l1N-ts1^*, a temperature sensitive allele of *N*, did not show conspicuous expansion of the ee population for 11-day-old flies cultured under a non-permissive condition (29°C) for *N^l1N-ts1^*, the double homozygous mutants for *βint-ν^2^* and *N^l1N-ts1^* showed its considerable expansion (Figure 4J–L). This indicates that *βint-ν* genetically interacted with Dl/N signalling factors in choice of cell fate between the ECs and ees as well as between the ISCs and EBs.

### Spread of EC misdifferentiation and ISC overproliferation from local to entire region in young *βint-ν* mutants

**Figure 5:**
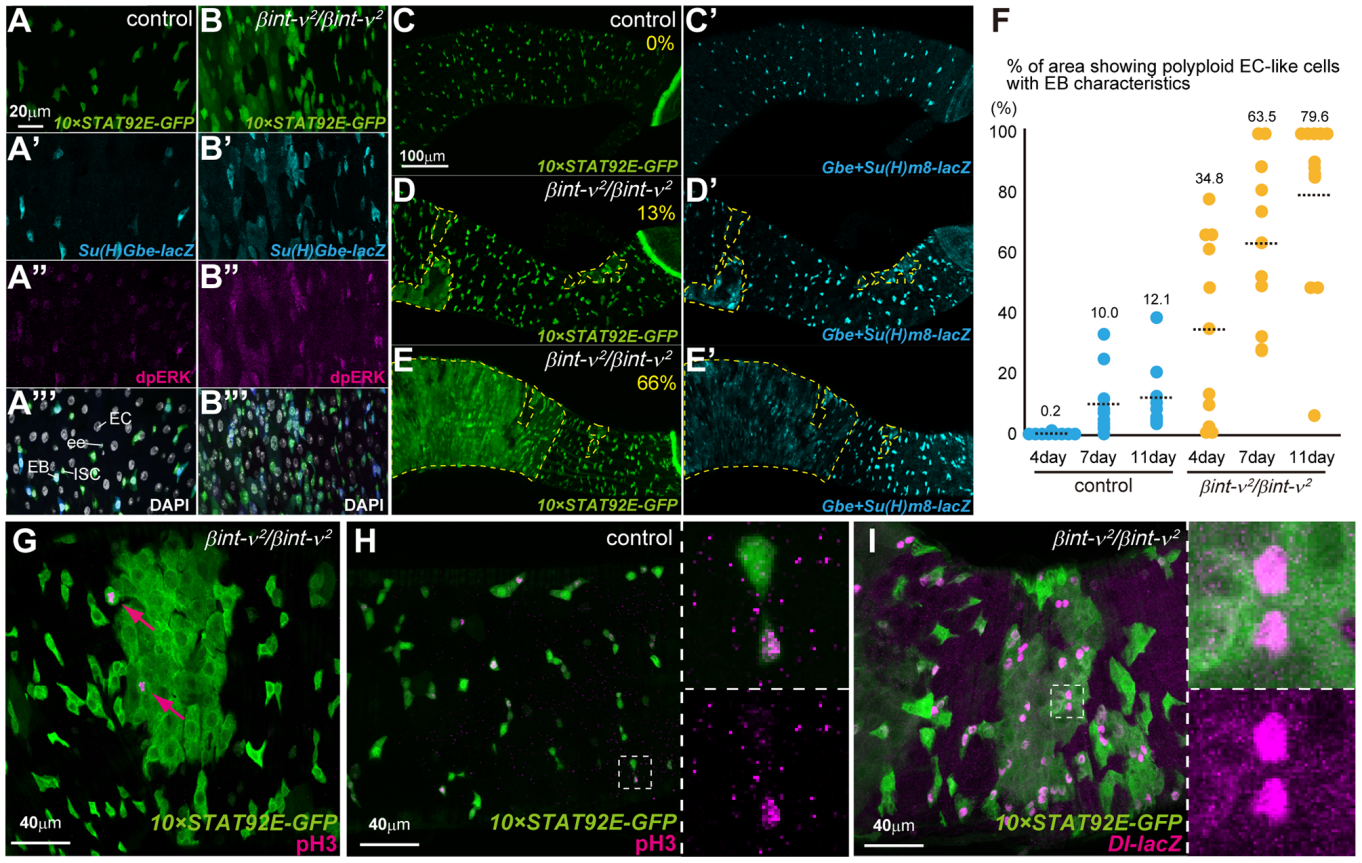
Misdifferentiated EC-like polyploid cells appear in young *βint-ν* mutants. (A–B) The PMG epithelial cells of 7-day-old control (A) and *βint-ν^2^* (B) flies, marked with *10×STAT92E-GFP* (green), *Su(H)Gbe-lacZ* (cyan), anti-dpERK antibody (magenta), and DAPI staining (white). (C-E) Examples of the PMG of control (*Su(H)Gbe-lacZ*/+, *10×STAT92E-GFP*/+) and *βint-ν^2^* (*Su(H)Gbe-lacZ*/+, *βint-ν^2^*/*βint-ν^2^*, *10×STAT92E-GFP*/+) flies showing *10×STAT92E-GFP* (green) and *Su(H)Gbe-lacZ* (cyan). The broken lines and percentages respectively indicate the outline of the region showing misdifferentiated EC-like polyploid cells expressing *10×STAT92E-GFP*+*Su(H)Gbe-lacZ* and the ratio of its area (measuring/calculation is detailed in the “Materials and methods” section). (F) Ratio of the abnormal epithelial area as indicated by broken lines in C–E for each of the control flies and *βint-ν^2^* homozygotes at 4, 7, and 11 days old. Each blue and orange circle represents a datum derived from a single PMG. Dotted lines and values indicate averages. (G) The local midgut epithelium with a patch of the misdifferentiated EC-like cells marked by *10×STAT92E-GFP* (green) in 7-day-old *βint-ν^2^* flies (*βint-ν^2^*/*βint-ν^2^*, *10×STAT-GFP*/+). In this region, pH3-positive ISC (magenta arrows) were predominantly contained. (H and I) The PMG of 7-day-old control (*10×STAT-GFP*/*Dl^05151^*) and *βint-ν^2^* (*βint-ν^2^*/*βint-ν^2^*, *10×STAT-GFP*/*Dl^05151^*) flies. The right panels are magnifications of the boxed areas in the left panels. Magenta staining in H and I represent pH3-positive and *Dl-lacZ*-positive cells, respectively.

In some reports, ISC overproliferation is often associated with induction of misdifferentiated EC-like polyploid cells that are continuously expressing EB markers [12], [13],[16]. To determine whether these misdifferentiated EC-like cells appeared in *βint-ν* mutants, we examined the expression of ISC and EB markers, i.e. *10×STAT92E-GFP*, *Su(H)Gbe-lacZ,* and a dual-phosphorylated form of ERK (dpERK), that had respectively been used as monitors for JAK/STAT, N, and EGF signalling activities. In control midguts of 7 day-old flies, *10×STAT92E-GFP* (strong in ISCs and EBs and weak in ees and VMs), *Su(H)Gbe-lacZ* (specific to EBs), and dpERK (ISCs, EBs, and a few ECs) were detected as previously reported (Figure 5A) [12], [16], [39]. On the other hand, in all the 7-day-old *βint-ν^2^* homozygotes, their atypical expressions in the EC-like polyploid cells were observed although their levels were weaker than their typical expressions in the ISCs and/or EBs (Figure 5B). These aberrant differentiation was randomly induced in patches of cell populations along the midgut at 7 days old, and rapidly spread throughout the young *βint-ν^2^* midguts with age (Figure 5C–F). In addition, mitosis and duplication of the ISCs expressing a high level of *Dl-lacZ* were preferentially observed in these abnormal region at 7 days old (Figure 5G–I). A similar differentiation defect was also induced by *βint-ν* RNAi in the ECs but not in the ISCs/EBs (Figure. S4). In addition, no obvious differentiation defects were observed in 1-day-old young flies and 96 hours after pupation (Figure. S5). Collectively, these observations suggested that abnormal EC-like polyploid cells, related to the ISC overproliferation, were induced during the young adult stage of *βint-ν^2^* flies and that *βint-ν* was essential for maintaining normal adult midguts but not for developing midguts.

### Local up-regulation of Upds was induced in young *βint-ν* mutants

**Figure 6:**
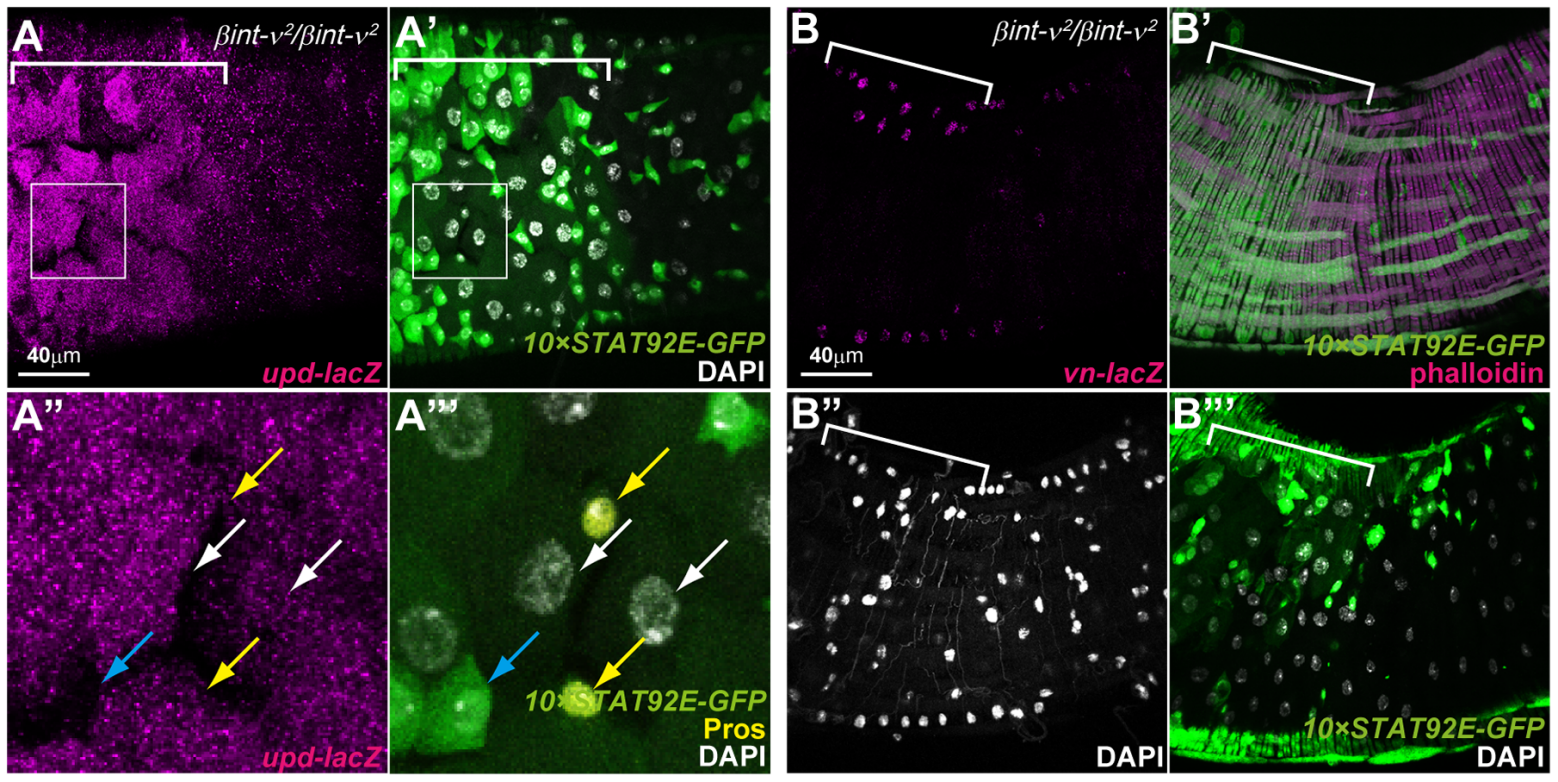
Induction of *upd* in epithelium and *vn* in VMs is initiated around the defective areas. (A–B) The PMG of 7-day-old *βint-ν^2^* homozygotes carrying *10×STAT92E-GFP* and *upd-lacZ* (A) or *vn-lacZ* (B). (A) The *upd-lacZ* (magenta in A and A”) was up-regulated in the polyploid EC-like (white arrows in A” and A”’) and the Pros-positive ee (yellow arrows in A” and A”’) cells weakly expressing *10×STAT92E-GFP* but not in the cells strongly expressing it (i.e. ISCs/EBs, cyan arrows in A’’ and A’’’). (B) The *vn-lacZ* (magenta in B) and *10×STAT92E-GFP* (green in B’) were up-regulated in the VMs, marked by phalloidin (magenta in B’), which surrounds the defective EC-like cell area (B”’). Brackets (in A, A’ and B-B”’) and squares (in A’’ and A’’’) indicate the regions of defective cells and high magnification (in A and A’), respectively. Nuclei were stained with DAPI (white in A’, A’’’, B”, and B”’). The genotypes in A-A”’ are *upd-lacZ*/+, *βint-ν^2^*/*βint-ν^2^*, and *10×STAT92E-GFP*/*+*, and those in B-B”’ are *βint-ν^2^*/*βint-ν^2^* and *10×STAT92E-GFP*/*vn-lacZ*.

In a stressed/damaged condition, Upds and Vn (ligands of JAK/STAT and EGF signalling pathways, respectively) are up-regulated in the ECs and VMs, which non-autonomously promotes ISC proliferation and differentiation [12], [13], [40]. In this study, we found that they were also up-regulated in the ECs/ees and VMs of *βint-ν* mutants on the basis of the expression of the reporter construct lines *upd-lacZ* or *vn-lacZ* (Figure 6A and B). The regions where this up-regulation was found corresponds to those in which the misdifferentiated EC-like cells were appeared (Figure 6A and B). In addition, we detected hyperactivation of JAK/STAT signalling in the VMs that surrounded the misdifferentiated ECs and up-regulated *vn-lacZ* (Figure 5B brackets). Combining these findings with information in previous reports, we speculated that in *βint-ν^2^* homozygotes the Upds secreted from defective EC-like cells promoted a local ISC proliferation and misdifferentiation of ECs by the direct pathway (from ECs into ISCs/EBs) and indirect pathway (from ECs into ISCs via VMs) where JAK/STAT signalling was activated by Upds from the ECs). In the latter case, JAK/STAT in the VM cells was known to induce an expression of *vn* that promoted ISC proliferation [41].

### *βint-ν* mutant phenotypes were suppressed by blockade of JNK signalling

**Figure 7:**
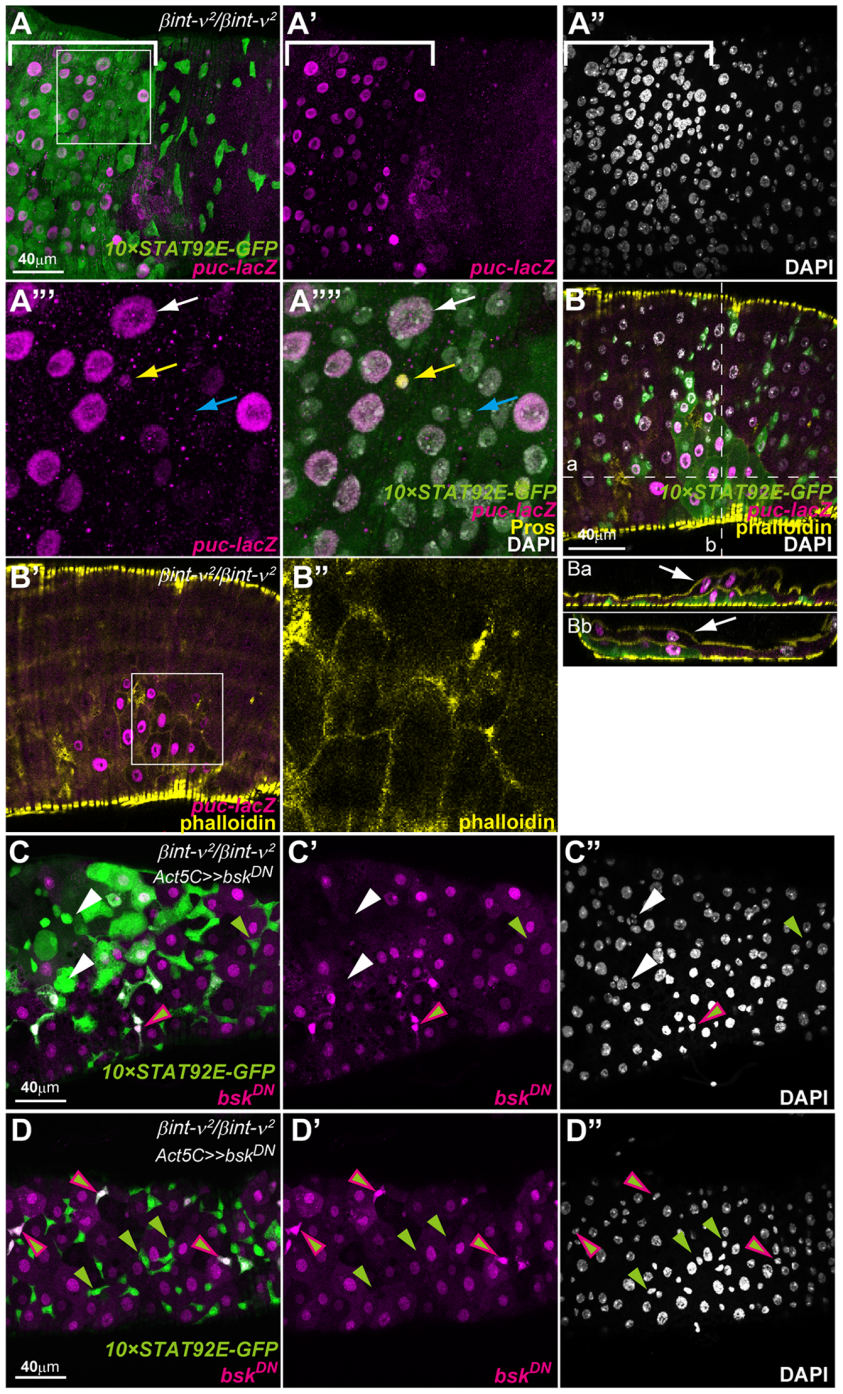
*βint-ν* mutant phenotypes were suppressed by inactivation of JNK signalling. (A-B) The PMG of 7-day-old *βint-ν^2^* homozygotes carrying *10×STAT92E-GFP* and *puc-lacZ* (*βint-ν^2^*/*βint-ν^2^*, *10×STAT92E-GFP*/*puc^E69^*). Nuclei were stained with DAPI (white). (A) In the region of the defective misdifferentiated ECs (brackets in A-A”), *puc-lacZ* (magenta) was up-regulated (A and A’) and an increase in cell number was observed (A”). (A”’ and A””) High magnifications of the boxed area in A. The up-regulation of *puc-lacZ* expression (magenta) was induced in the polyploid EC-like cells (white arrow) and Pros-positive ee cells (yellow arrow) but not in the other diploid cells (cyan arrow). (B-B”) The F-actin (yellow) was accumulated at the outlines of cells showing high level activation of JNK signalling (B’ and B”), in which two-layered epithelium was observed (arrows in Ba and Bb). B” is a high magnification of the boxed area in B’, and Ba and Bb are Z-sections indicated by broken lines in B. (C–D) The PMG of *βint-ν^2^* homozygotes with a *bsk^DN^*–expressing mosaic obtained with a FLP-out system (*hs-flp*/*UAS-bsk^DN^*, *βint-ν^2^*/*βint-ν^2^*, *10×STAT92E-GFP*/*GAL4-Act5C(FRT.CD2)*, *UAS-RFP*). (C) Misdifferentiated cells (with both *10×STAT92E-GFP* and polyploid nucleus) and signs of overproliferation (white arrowheads) were observed in the regions without *bsk^DN^*–expressing EC-like polyploid cells (upper left area). (D) No defects were caused in the regions where *bsk^DN^* was expressed in the polyploid ECs. The ISC/EB-like diploid cells, circumscribed by ECs expressing *bsk^DN^*, showed a normal aspect in appearance, irrespective of the presence (green-magenta arrowheads) or absence (green arrowheads) of *bsk^DN^* expression.

It has been found that JNK signalling, known as a stress/damage sensor, is dependent on the defect levels in the ECs. It promotes ISC proliferation and differentiation to rapidly regenerate the epithelium via interleukin-6-like secreted factors Unpaired (Upds) [19], [42], [43]. It has also been reported that hyperactivation or inactivation of JNK signaling in ISCs/EBs affected proliferation of ISCs that resulted in reducing shortened lifespan [44]. From these reports and our result on *βint-ν* mutant midguts showing up-regulation of *upd-lacZ*, we speculated that activation of JNK signalling accompanied the up-regulation of Upds. To monitor JNK signalling activity, we used *puc^E69^* (*puc-lacZ*), a *lacZ* reporter line [45]. As expected, *βint-ν^2^* homozygotes showed a high level activation of JNK signalling in the EC-like polyploid cells and Pros-positive ee cells around the regions where the misdifferentiated EC-like cells were induced (Figure 7A and B). In these regions, a local increase in the cell number (Figure 7A”), two layered epithelia (arrows in Figure 7Ba and Bb), and an accumulation of F-actin (Figure 7B’ and B”) were also detected clearly in *βint-ν^2^* homozygotes carrying *puc^E69^*. Since *puc^E69^* is a loss of function allele of *puc* that encodes a negative regulator of JNK signalling, heterozygosity of this allele results in a slightly high level of JNK signalling. Accordingly, the above phenotypes were probably enhanced when compared with those observed in normal *puc* condition.

To further examine whether the excess ISC proliferation and EC misdifferentiation in *βint-ν^2^* homozygotes were dependent on JNK signalling, we performed mosaic suppression of JNK singalling by forced expression of *bsk^DN^*, a dominant negative form of *bsk*, in *βint-ν^2^* homozygotesby the FLP-out system [46]–[48]. Although signs of overproliferation and misdifferentiation were obvious in the regions without *bsk^DN^*, they were not observed in the region with *bsk^DN^* in most of the ECs (Figure 7C, out of 10 midguts observed). Remarkably, all of the ISC- and EB-like diploid cells with or without *bsk^DN^* showed normal aspects in the regions surrounded by ECs expressing *bsk^DN^* (green and green+magenta arrowheads in Figure 6C and D). This suggested that the overproliferation of ISCs and misdifferentiation of ECs in *βint-ν* mutants depended on up-regulation of JNK signalling in the ECs.

### Local defects in young *βint-ν* ^2^ mutants were not directly related to cell death

Previous reports showed that activation of JNK signalling induced apoptosis in the ECs [42]. Therefore, we performed TUNEL staining for the *βint-ν* ^2^ mutant midguts. Although a slight increase in the dying ECs was detected in the *βint-ν* ^2^ mutants, compared with wild type, at 14 days old (Figure S6A and B), no common cell death processes (e.g. fragmented nuclei, cell blebbing, sloughing of cells into the lumen) were obtained with cross-section analyses (Figure. 2D and data not shown). In addition, no TUNEL-positive cells were observed in the local defective regions with ectopic JAK/STAT activation in the *βint-ν* ^2^ midgut (Figure S6C). Thus the slight increase in cell death observed at 14 day old was probably a secondary effect in the *βint-ν* ^2^ midgut.

## Discussion

### *βint-ν* represses senescence of the adult midgut epithelium

**Figure 8:**
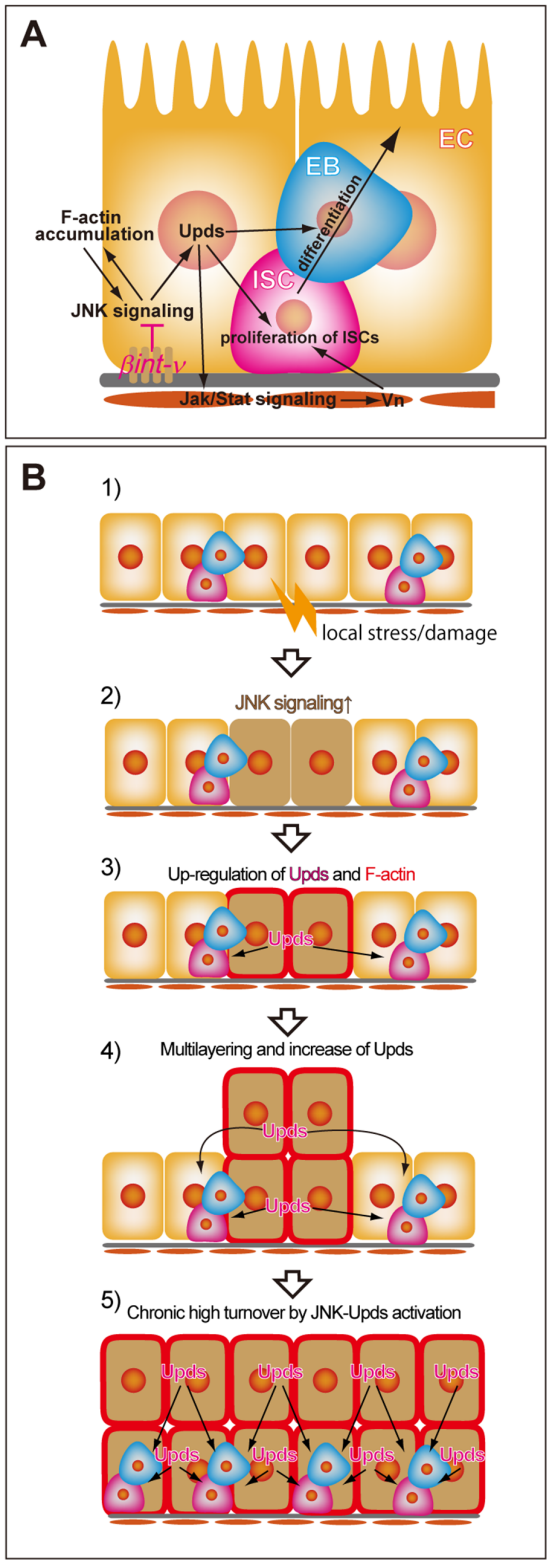
A model for homeostasis disruption in *βint-ν* mutant. (A) A schematic diagram of the *βint-ν* signalling pathway for regulating ISC proliferation and differentiation. *βint-ν* inhibits up-regulation of JNK signalling in young flies cultured under normal condition. The up-regulation of JNK signalling induced *upd* transcription as well as F-actin accumulation. Upds in turn promoted ISC proliferation (duplication) and differentiation. In addition, accumulation of F-actin related to activation of JNK signalling. (B) A possible model for progression of homeostasis disruption of the adult midgut epithelium in *βint-ν* mutants. 1) Locally and weakly stressed/damaged cells are produced. 2) JNK signalling is activated. 3) Upds and F-actin are up-regulated, which promotes ISC proliferation (duplication) and differentiation as shown in D. 4) Retention of JNK–active cells and F-actin-mediated multilayering results in a further increase in Upds. 5) Expansion of epithelium with a high turnover rate from the local defective region (step 4) together with additional local stress/damage (step 1) cause an entire and chronic high level activation of the JNK-Upds pathway for additional defects such as midgut shortening.

A few studies have reported that *βint-ν* was involved in the embryonic endoderm migration and phagocytosis for apoptotic cells [29], [30], [49]. In this study, we found a novel function of *βint-ν* in maintaining homeostasis of the adult midgut epithelium. The adult midgut of *βint-ν* mutants exhibited a shortened and multilayered epithelium, which became more severe with aging (Figure 1). Deterioration of these defects are probably due to increases in the ISCs and misdifferentiated EC-like cells (Figures 2–4). Genetic manipulation for several signalling pathways, bacterial infections, or spontaneous aging have also been reported to cause excess ISC proliferation and abnormal cell differentiation accompanied by shortening and multilayering of the epithelium. [10], [40], [41]. Thus, maintaining the morphology of the *Drosophila* adult midgut is considered to be closely linked to proper ISC proliferation and differentiation. These processes are known to be regulated by several signalling pathways including Notch, JAK/STAT, EGF, and JNK signalling. The *βint-ν* also inhibits misregulation of these signalling pathways induced by hyperactivation of JNK signalling as discussed below (Figure 8A) to maintain normal ISC proliferation and differentiation that ensures homeostasis of the midgut morphology and cell type proportion.

### *Mys* and *βint-ν* probably have different roles in the adult midgut epithelium

Via integrins, the ISCs adhere to the VMs that provide niche factors such as Wg, Vn, and Dilp3, promoting the ISC proliferation [15], [36], [50]. The other *Drosophila* integrin β subunit gene, *mys*, is required for maintenance of germline and follicle stem cells to anchor the niche [25], [26]. Most recently, Goulas et al. reported that *mys* was also involved in asymmetric ISC division [27]. In addition, *mys* RNAi in ISCs and ECs respectively resulted in an increase in and no effect on ISC proliferation [27]. However, we obtained a different result, i.e. that *mys* RNAi in the ISCs/EBs with *esg-*GAL4 resulted in decreasing the ISCs/EBs (Figure S7A, S7C and 3K) and delaying growth of the ISC-like tumour induced by *N* RNAi (Figure S7F compared with S7D) as most recently reported by Lin et al [28]. These suggested that, unlike *βint-ν*, *mys* was required for maintaining the ISCs. By contrast, *mys* RNAi in the ECs with *MyoIA*-GAL4 caused an increase in mitotic ISCs and multilayered epithelia, which were more severe than that caused by *βint-ν* RNAi (Figure 3J, 3M-O and data not shown). Thus, according to our results, in the ISC proliferation/maintenance, *mys* played opposite roles between cell types, that is, a role in the ISCs/EBs essential for maintaining the ISCs and a role in the ECs essential for suppressing the ISC proliferation. On the other hand, *βint-ν* phenotypically showed its function only in the ECs for the ISC proliferation (Figures 2 and 3). Consistently, the *βint-ν* mutants did not show any alteration of Mys localization in the midgut (Figure S8). In tumour progression of vertebrates, some integrins such as αvβ3 and α6β4 enhance the progression, whereas others such as α5β1 inhibit it [21]. It is thought that one possible reason for these differences is that the expression levels in each integrin are varied with cell types.

### Role of JNK activation to suppress midgut scenescence

In young *βint-ν* mutants, hyperactivation of JNK signalling was induced in the EC-like and ee cells just around the local defects with ISC proliferation and EC misdifferentiation, which were suppressed by inactivation of JNK in the ECs (Figure 7). It has been hypothesized that, in response to local tissue damage, activation of JNK signalling in the ECs non-autonomously promotes ISC proliferation and differentiation via Upds, which also induces *vn* expression through JAK/STAT activation in the VMs [41]. Consistently with this finding, in our observation, up-regulation of *upd* in the EC-like and ee cells and *vn* expression in the VMs also occurred in the defective region (Figure 6). Therefore, we can interpret that the normal *βint-ν* gene product inhibits hyperactivation of the JNK-Upds pathway, which nonautonomously induces ISC overproliferation and EC misdifferentiation via direct (ECs into ISCs/EBs) and indirect (mediated with VMs) pathways (Figure 8A).

For example, overproliferation, misdifferentiation, and high cell turnover state were provoked together in patches of cell populations in the midgut challenged by *Ecc15*, a non-lethal strain of the phytobacteria *Erwinia carotovora* [43]. In this case, clustered apoptotic cells accompanied by highly activated JNK signalling in the ISCs/EBs/ECs were also induced to improve the midgut shrinkage, an acute symptom of bacterial infection [40], [43]. Thus, the local, transient, and high level activation of JNK was important in regenerating the midgut epithelium.In the case of young *βint-ν* mutants, similar local defects such as high cell turnover and high level (but not transient) JNK activation in the EC-like /ee cells (Figure 5 and 7) were observed, which led us to the idea that the state of *βint-ν* mutant midgut mimics the stressed/damaged conditions such as infection by bacteria (Figure 8B). However, unlike the case of bacterial infection, the defects in the young *βint-ν* mutants rapidly spread throughout the midgut and the midgut shortening was never restored later (Figure 2). On the other hand, the normally aged wild type midgut has also been found to show a high cell turnover with chronic high level activation of JNK signalling in the ECs and ees throughout the midgut [10]. Also in aged midgut, *esg-GAL4* was induced in misdifferentiated EC-like cells like the ectopic expression of *10×STAT92E-GFP* in young *βint-ν* mutant midgut [44]. In this condition, artificial activation of JNK signalling accelerated overproliferation of ISCs, which accompanied short lifespan [44]. Thus, the symptoms of the *βint-ν* mutant midgut were more similar to those of an aged midgut than a bacterially infected midgut, and *βint-ν* probably plays two roles through its inhibiting of JNK. One is suppressing random causes of the local high cell turnover. The other is inhibiting the spread of the defects, which subsequently leads to chronic disorganization of the epithelial cell turnover (Figure. 8B).

In the local defects, accumulation of F-actin, which is related to JNK signalling, was observed at the cell-cell boundaries (Figure 7B). We are considering that this is also related to the local increase of ISC proliferation (data not shown). Additionally, abnormally two-layered epithelia were observed only in the local areas with F-actin accumulation (Figure 7B), suggesting that this leads to inhibition of epithelial elimination or promotion of EC misdifferentiation. Furthermore, *Hippo* signalling might be involved in mediating JNK signalling activity and F-actin dynamics in the midgut defects of *βint-ν* mutant, since the *Hippo* pathway functions as a known sensor of cell adhesion and cytoskeletal integrity in the ECs to regulate ISC proliferation and promote it to a stress-response condition via Upds [18], [19], [34], [51]–[53].

Fate asymmetry of the ISC offsprings, ISC self-renewal, and EB commitment are required for ensuring a constant ISC pool. The fate choice is known to be regulated through lateral inhibition by Dl/N signalling between the neighboring ISC siblings, and its ratio is also affected by proximate differentiated cells such as the damaged ECs [38]. Our study indicated that frequent ISC duplication was induced in *βint-ν* mutants (Figures 4 and 5), which suggested that the normal *βint-ν* gene product in the ECs nonautonomously affected Dl/N interaction between the ISCs through the JNK-Upds pathway (Figure 8A). Furthermore, the high level of *Dl-lacZ* expression and change in D1 localization observed in *βint-ν* mutants suggested that the expression level and/or D1 localization might be involved in the fate choice of the ISC offsprings (Figure 4).In previous reports, aging and hyperactivation of JNK signalling also affected N signalling and ISCs proliferation [10], [44]. Future studies should provide more understanding of the molecular mechanisms for switching between fate symmetry/asymmetry of the ISC offsprings in response to various midgut conditions.

### Integrins are possibly involved in age-related disease caused by disruption of tissue homeostasis

In young *βint-ν* mutants, hyperactivity of JNK-Upds signalling led to chronic hyperplasia of the ISCs throughout the midgut. As discussed above, this symptom was similar to that of aged phenotypes of the midgut [10], [54], [55]. Therefore, we speculated that *βint-ν* probably inhibits premature aging of the *Drosophila* adult midgut. There is much evidence suggesting that aging creates conditions conducive to the incidence of neoplastic diseases such as cancer [56]. In mammals, the cytokine interleukin-6 and its downstream effector STAT3, a homologous pathway of the *Drosophila* Upds/JAK/STAT signalling, are involved in inducing intestinal inflammation and cancer [57], [58]. Together with Wnt signalling, JNK signalling activation also leads to tumour formation in the vertebrate intestine [59]. Furthermore, moderate activation of JNK resulted in an increased tolerance to stresses and extended lifespan but its excessive or chronic activation was implicated in the occurrence of pathologies including cancer [60]. Although the relationship between integrin dysfunction and progression of aging in mammals remains unclear, our results indicate that the chronic activation of JNK and subsequent elevation of cytokines caused by integrin dysfunction were likely involved in inducing or affecting age-related diseases. These findings provide an insight into a possible etiology of age-related diseases such as colorectal carcinoma that may be affected by dysfunction of the integrin function as seen in the above-mentioned case of integrin α5β1 [21].

## Materials and Methods

### Fly strains

We used *w^1118^* as a wild-type strain, as well as *βint-ν* ^2^, which is a null allele of *βint-ν* [29]. The following mutant and reporter lines were used: *N^ 1N-ts1^* (a temperature sensitive allele of *N* [61], *Dl^05151^* (*Dl-lacZ*), *PD* (*upd-lacZ*), *vn^10567^* (*vn-lacZ*), *puc^E69^* (*puc-lacZ*), and *esg^k00606^* (*esg-lacZ*) (lacZ enhancer trap lines of *Delta*, *unpaired*, *vein*, *puckered*, and *escargot*, respectively [10], [45], [62]–[65], *Su(H)Gbe-lacZ* and *10×STAT92E-GFP* (reporter lines of Notch and JAK/STAT signalling activity, respectively [66], [67], and *vkg-GFP* (a protein trap line expressing green fluorescent protein (GFP) fused to Viking, the *Drosophila* Collagen IV [8]). The GAL4 lines used were *NP6267* (*esg-GAL4*) [68], *NP1* (*MyoIA-GAL4*) [12], *how^24B^* (*24B-GAL4*) [69], *NP0319* (103585), *Ay-GAL4* [70], and *Act5C-GAL4* (*FRT.CD2*) [71]. The following UAS strains were used: *UAS-GFP^S65T^*and *UAS-RFP.nls* obtained from Bloomington *Drosophila* Stock Center (BDSC), *UAS-bsk^DN^* (a dominant negative form of *basket*, the *Drosophila* JNK [46]), *UAS-βint-ν* RNAi (#40895 from Vienna *Drosophila* RNAi Center (VDRC)), *UAS-mys* RNAi (#29619 from VDRC), and *UAS-N* RNAi (#100002 from VDRC). The *hs-FLP* provides FLP recombinase under the heat-shocked condition. The *tub-GAL80^ts^* ubiquitously overexpresses a temperature-sensitive GAL80 protein [35]. We obtained the tub-GAL80, hs-FLP, w^*^, FRT19A and the *FRT19A* strains used for MARCM analysis from BDSC.

Flies were cultured in standard medium and at appropriate temperature and female individuals were observed in all the experiments in this study.

### Immunostaining and in situ hybridization

A dissected adult midgut at an appropriate adult stage was fixed with 4% paraformaldehyde. After it was washed with phosphate buffered saline containing TritonX-100, immunostaining was performed with the following primary antibodies: rat anti-βint-ν (1∶200) [30], rabbit anti-βint-ν (1∶200) [24], rat anti-GFP (Nacalai Tesque, 1∶200), rabbit anti-RFP (Clontech, 1∶200), chick anti-β-galactosidase (β-gal) (abcam, 1∶200), rabbit anti-phospho-histone H3 (pH3) (Upstate Biotech, 1∶200), mouse anti-dpERK (Invitrogen), mouse anti-γ-tubulin (GeneTex, 1∶100), mouse anti-Prospero (DSHB, 1∶100), mouse anti-Armadillo (DSHB, 1∶50), mouse anti-Myospheroid (DSHB, 1∶100), and mouse anti-Delta (DSHB, 1∶50). Secondary antibodies used were Cy3- or DyLight649-conjugated anti-mouse IgG (Jackson ImmunoResearch, 1∶200), Alexa488-conjugated anti-rat IgG (Jackson Immuno Research, 1∶200), Cy2- or Alexa Fluor555-conjugated anti-rabbit IgG (Jackson ImmunoResearch, 1∶200), Alexa647-conjugated anti-rabbit IgG (Jackson ImmunoResearch, 1∶200), and DyLight649-conjugated anti-chick IgY (Jackson ImmunoResearch, 1∶200). Rhodamine-conjugated and Alexa Fluor 647-conjugated phalloidin (Molecular Probe, 1∶100) were used to stain F-actin. Nuclei were stained with 4′6-diamidino-2-phenylindole (DAPI, SIGMA) or TOTO-3 (molecular probes).

In situ hybridization was performed by standard protocol with anti-Digoxygenin labelled with alkaline-phosphatase (Roche, 1∶7000) and Fast Red (Roche). We used cDNAs of *βint-ν* as templates to perform antisense and sense RNA probe synthesis with a DIG RNA labelling kit (Roche).

The stained midgut was mounted in 80% glycerol and analysed with Nikon Digital Eclipse C1 and C1Si confocal microscopes (Nikon). The images were processed with EZ-C1 3.90 Free Viewer software (Nikon).

### TUNEL assay

The dissected adult midgut was fixed with 4% paraformaldehyde and cell death was detected with an ApopTag Kit (Millipore Corporation, Billerica, MA, USA).

### Experiments with temperature shift

When knockdown and overexpression experiments with a TARGET system [35] were performed, adult flies of a given genotype that were carrying *tub-GAL80^ts^* were raised at 18°C, a permissive temperature. One-day-old flies were placed and cultured in a non-permissive condition (29°C) for an appropriate period before dissection.

We used the FLPout technique [47], [70] to raise and culture other adult flies at 18°C until they were 7 days old. These flies were heat-shocked at 37°C for 20 minutes and cultured at 25°C for an appropriate number of days.

We raised *N^ 1N-ts1^*/*N^ 1N-ts1^*, *βint-ν* ^2^/*βint-ν* ^2^, and *N^ 1N-ts1^*/*N^ 1N-ts1^*, *βint-ν* ^2^/*βint-ν* ^2^ females at 18°C until eclosion and cultured them at 29°C for 11 days.

In MARCM clone generation [37], control (*FRT19A, tub-GAL80, hs-FLP/FRT19A, Act5C-GAL4, UAS-GFP^S65T^/+*) and *βint-ν* ^2^ *(FRT19A, tub-GAL80, hs-FLP/FRT19A, βint-ν^2^/βint-ν^2^, Act5C-GAL4, UAS-GFP^S65T^/+*) flies were raised and cultured at 25°C until they were 7 days old, heat-shocked 4 times at 37°C for 2 hours and additionally cultured for 5 days.

### Measurements, cell counts, and statistical analysis

We dissected midguts out from five wild-type flies and five *βint-ν* ^2^ homozygote flies, at each age, in order to measure their lengths. After DAPI staining, we used confocal microscopes to obtain images of the whole midguts, then used ImageJ (NIH) software to process and measure them.

After preparing the whole stained midguts with an anti-pH3 antibody, a fluorescence microscope was used to count the pH3-positive cells (mitotic cells) in them.

To measure the angle between spindle and basal surface, 14 (wild-type) and 20 (*βint-ν* ^2^ homozygote) 7-day-old mitotic cells from prometaphase to anaphase in the posterior midgut (PMG) were stained with anti-γ-tublin and anti-pH3 antibodies and phalloidin.

To calculate the ratio of areas showing misdifferentiated cells (polyploid nuclei, *10×STAT92E-GFP*, and *Su(H)Gbe-lacZ-*positive EC) in the PMG, control (*Su(H)Gbe-lacZ*/+, *10×STAT92E-GFP*/+) and *βint-ν* ^2^ homozygote (*Su(H)Gbe-lacZ*/+, *βint-ν* ^2^/*βint-ν* ^2^, *10×STAT92E-GFP*/+) flies at each age were dissected, then stained with anti-GFP and anti-β-gal antibodies. One-side images of them were then obtained with the 20×objective lens of a confocal microscope, with a focus on the boundary between the midgut and hindgut, as shown in Fig. 4C–E. The total differentiation defect area was measured with ImageJ (NIH) software. The average ± S.D. of the total area obtained was 94653±17209dpi (control at 4 days old, n = 10), 103802±22616dpi (control at 7 days old, n = 11), 135312±32651dpi (control at 11 days old, n = 10), 104397±12674dpi (*βint-ν* ^2^ at 4 days old, n = 11), 90862±33413dpi (*βint-ν* ^2^ at 7 days old, n = 11), and 105521±17078dpi (*βint-ν* ^2^ at 11 days old, n = 12) with resolution set to 1024×1024 dpi.

The Student's t-test was used to calculate P-values as needed. We also used the following software: EZ-C1 3.90 FreeViewer (Nikon), Photoshop (Adobe), and Illustrator (Adobe).

### Phylogenetic analysis

The predicted amino acid sequences encoded by the other *Drosophila* species's *βint-ν* and *mys* orthologous genes that were derived from the 12 *Drosophila* genome projects [72], [73] were retrieved from GenBank. However, the *D. simulans* and *D. persimilis* genes were not analyzed since the sequence data of C-terminal region including highly-conserved transmembrane domain of the *D simulans*'s *βint-ν* and *D. persimilis*'s *mys* were not obtained from the database even with BLAST analysis. Using the available predicted amino acid sequences, we constructed a multiple alignment by the program MAFFT (http://www.genome.jp/) using the default parameters. Based on this alignment, the phylogenetic tree was conducted using MEGA6 program by Neighbor-joining method. The bootstrap test was performed with 1000 replicates. The result obviously indicated the presence of two distinct group, *βint-ν* and *mys* (Figure S1).

## Supporting Information

Figure S1 **Phylogenetic analysis of the** ***βint-ν*** **and** ***mys*** **orthlogous genes.** A phylogenetic tree represents the relationship among *βint-ν* and *mys* orthologous genes of several *Drosophila* species. The tree shows an evolutionary conservation of two distinct groups, *βint-ν* and *mys*. The bootstrap values and gene names were indicated next to each branch point and in the parentheses, respectively.
(TIF)

Figure S2 ***βint-ν*** **RNAi in ECs did not affect BM.** (A and B) The PMG of control and *βint-ν* RNAi treatment with *MyoIA-GAL4* driver for 14 days. In the *βint-ν* RNAi midgut, any obvious defects of Vkg-GFP (green), which labeled a part of BM, were not detected (B), when compared with control (A). Circular visceral muscles (cvm) and longitudinal visceral muscles (lvm) stained with phalloidin (magenta) were also normal in control (A’) and *βint-ν* RNAi midgut (B’). Epithelial cells, of which nuclei were stained with DAPI (white), were overcrowded in *βint-ν* RNAi midgut (B”), when compared with control (A”).
(TIF)

Figure S3 **Angle between the spindle body and BM was frequently lower in mitotic ISCs of** ***βint-ν*** **mutants.** (A) An example of mitotic ISCs stained with anti-pH3 antibody (green), anti-γ-tubulin antibody (magenta), and phalloidin (yellow). θ is an angle between the spindle body and BM indicated with broken lines. (B and C) The graphs showing frequencies of the angle (θ) measured in the PMG of wild-type (B) and *βint-ν^2^* homozygous mutants (C). Values shown at each bottom are average ± S.E.M.
(TIF)

Figure S4 ***βint-ν*** **RNAi in ECs but not in ISCs/EBs affected expression of** ***10×STAT92E-GFP*** **.** (A and A’) The PMG where *βint-ν* RNAi was performed with *MyoIA-GAL4* driver. Abnormal expression of *10× STAT92E-GFP* (green) was induced in the *βint-ν* RNAi midgut, compared with control (Figure 5A, C, and H). (B and C) The PMG where *βint-ν* RNAi was performed with *esg-GAL4* driver. Expression of *10×STAT92E-GFP* (green) was normal in both control (B) and *βint-ν* RNAi (C) flies. The *esg-GAL4*-driven expression was monitored with *UAS-RFP* (magenta in B’ and C’). Nuclei were stained with DAPI (white in B’’ and C’’).
(TIF)

Figure S5 **The midgut of** ***βint-ν*** **mutant did not show differentiation defects at the pupal stage and 1day-old adult stage.** (A–D”’) The PMG of control and *βint-ν* ^2^ homozygotes at 1day-old adult (A–B) and 96 hour after puparium formation (APF) (C–D). Expression pattern of *10× STAT92E-GFP* (green), *Gbe-Su(H)m8-lacZ* (cyan), and Pros (magenta) was not altered in *βint-ν* ^2^ midgut. Arrow in D indicates a signal from the yellow body in the gut lumen [74]). Nuclei were stained with DAPI (white).
(TIF)

Figure S6 **Faint increase of cell death in the regions with local defects caused in** ***βint-ν*** **mutant.** (A and B) The PMG of wild-type and *βint-ν* ^2^ homozygotes at 14day-old, where TUNEL assay was performed. In wild-type (A), strong TUNEL signal (magenta) was frequently undetected, but, in the *βint-ν* ^2^ mutants (B), a slight increase of TUNEL-positive cells was observed at 14-days-old. (C) At 7-day-old flies, TUNEL-positive cells did not appear in the region of the local defects with ectopic JAK/STAT activation (indicated by broken lines) caused in *βint-ν* ^2^ midgut. Nuclei were stained with DAPI (white).
(TIF)

Figure S7 ***mys*** **RNAi in ISCs/EBs affected maintenance of ISCs.** (A–C) The PMG with non-RNAi (control)(A and A’), *βint-ν* RNAi (B and B’), and *mys* RNAi (C and C’) treatment using *esg-GAL4* driver. In their treatments for 14 days, only *mys* RNAi caused a decrease in number of *esg*-positive cells (green). (D–F) The PMG with *N* RNAi (D and D’), *βint-ν* RNAi +*N* RNAi (E and E’), and *mys* RNAi +*N* RNAi (F and F’) treatments using *esg-GAL4* driver. Growth of ISC-like (green) and ee-like (magenta) tumor induced by *N* RNAi was inhibited by *mys* RNAi but not by *βint-ν* RNAi. Nuclei were stained with DAPI (white).
(TIF)

Figure S8 **Distribution of Mys in the PMG was not affected by** ***βint-ν*** **mutation.** (A–C) The PMG of wild-type at 14-day-old (A and A’), *βint-ν* ^2^ homozygote at 14-day-old (B and B’), and *βint-ν* ^2^ homozygote at 28-day-old (C and C’), stained with anti-Mys antibody (green) and phalloidin (magenta). Distribution pattern of Mys was not affected by *βint-ν* ^2^ homozygosity.
(TIF)
