# Supplementary figures and images for: βν Integrin Inhibits Chronic and High Level Activation of JNK to Repress Senescence Phenotypes in Drosophila Adult Midgut
Source: PLoS One. 2014 Feb 20;9(2):e89387. doi: 10.1371/journal.pone.0089387 (PMC3930726; doi:10.1371/journal.pone.0089387)

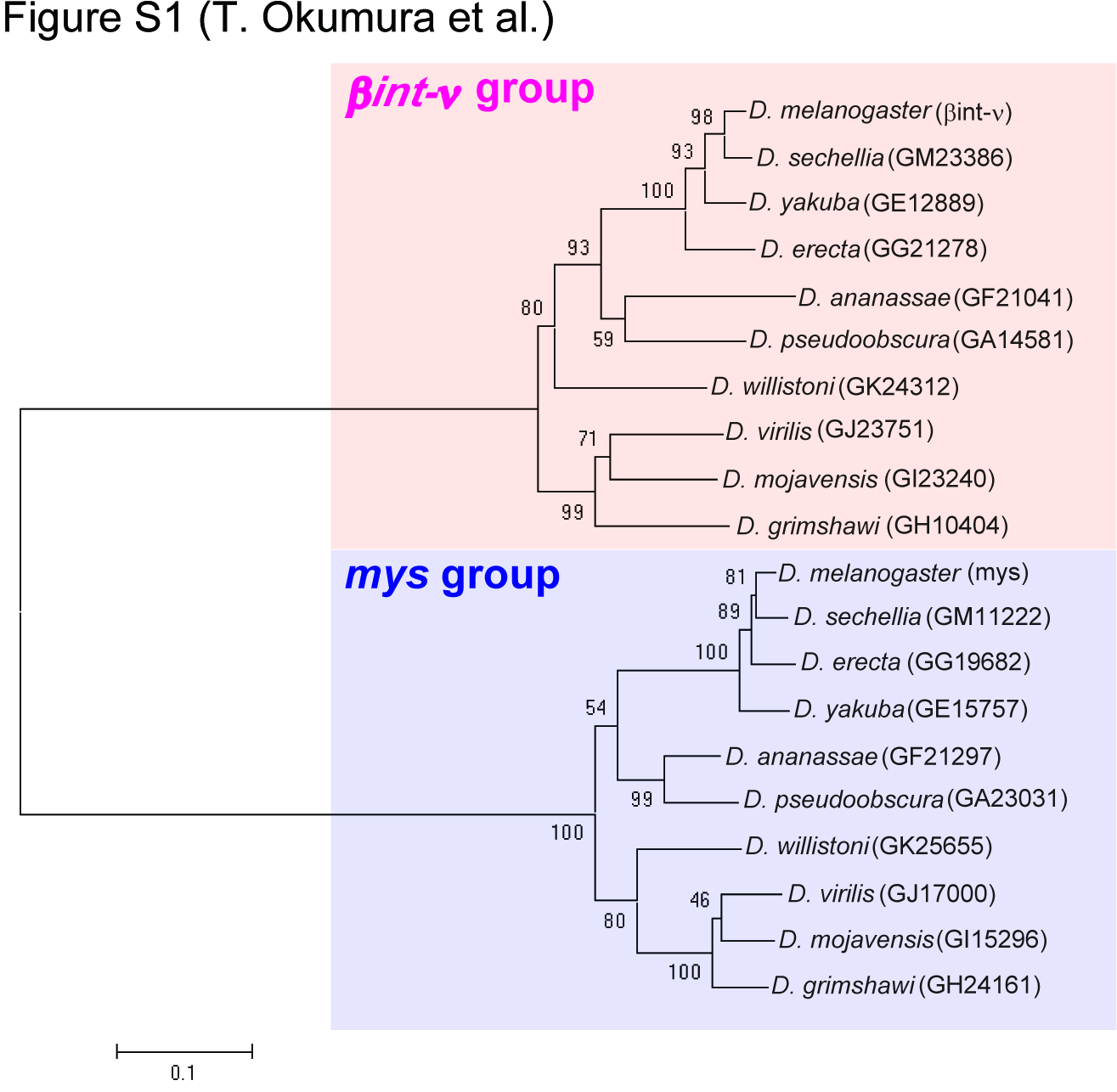

Supplement: Figure S1 — Phylogenetic analysis of the βint-ν and mys orthlogous genes. A phylogenetic tree represents the relationship among βint-ν and mys orthologous genes of several Drosophila species. The tree shows an evolutionary conservation of two distinct groups, βint-ν and mys. The bootstrap values and gene names were indicated next to each branch point and in the parentheses, respectively. (TIF) [file pone.0089387.s001.tif]

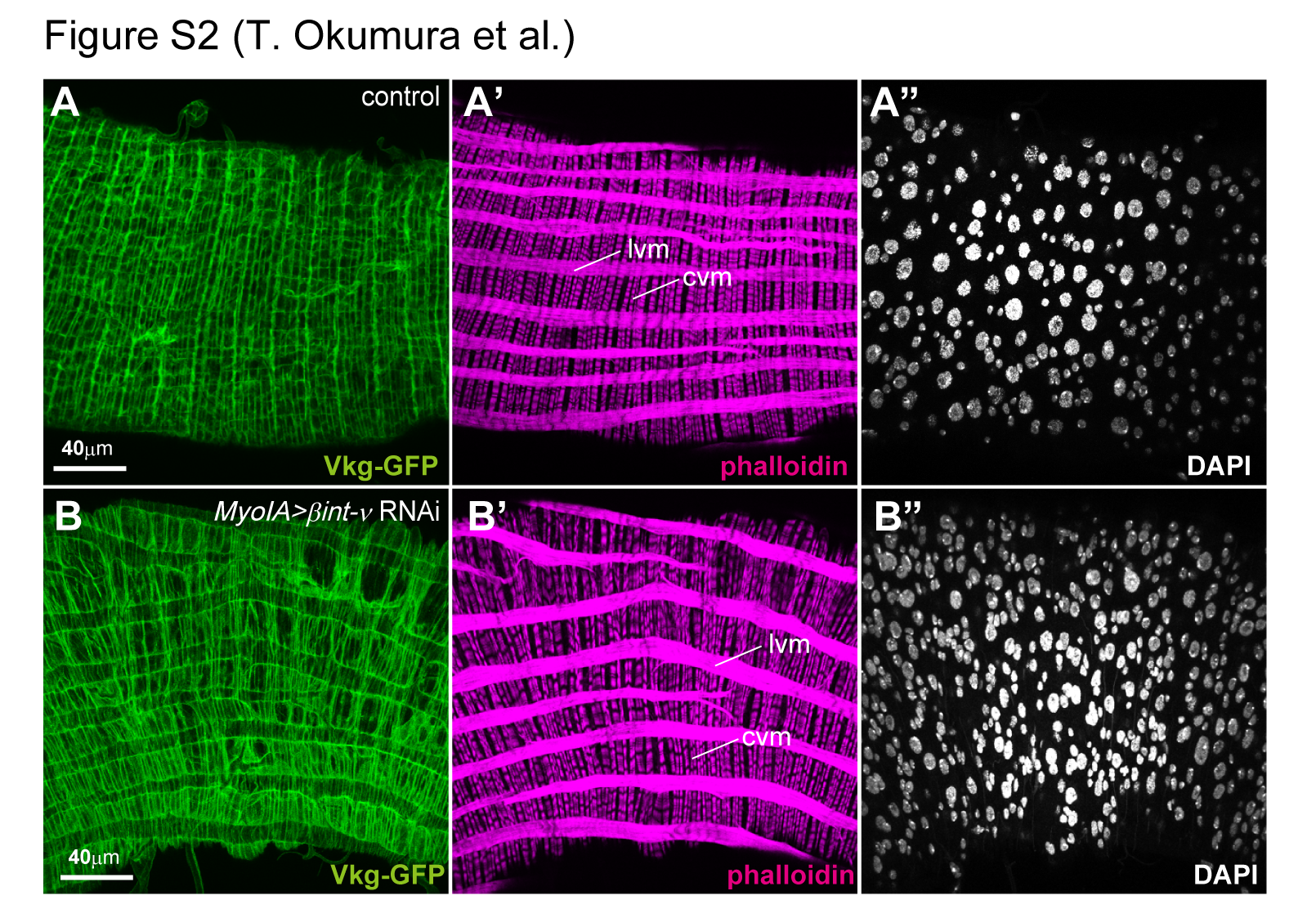

Supplement: Figure S2 — βint-ν RNAi in ECs did not affect BM. (A and B) The PMG of control and βint-ν RNAi treatment with MyoIA-GAL4 driver for 14 days. In the βint-ν RNAi midgut, any obvious defects of Vkg-GFP (green), which labeled a part of BM, were not detected (B), when compared with control (A). Circular visceral muscles (cvm) and longitudinal visceral muscles (lvm) stained with phalloidin (magenta) were also normal in control (A’) and βint-ν RNAi midgut (B’). Epithelial cells, of which nuclei were stained with DAPI (white), were overcrowded in βint-ν RNAi midgut (B”), when compared with control (A”). (TIF) [file pone.0089387.s002.tif]

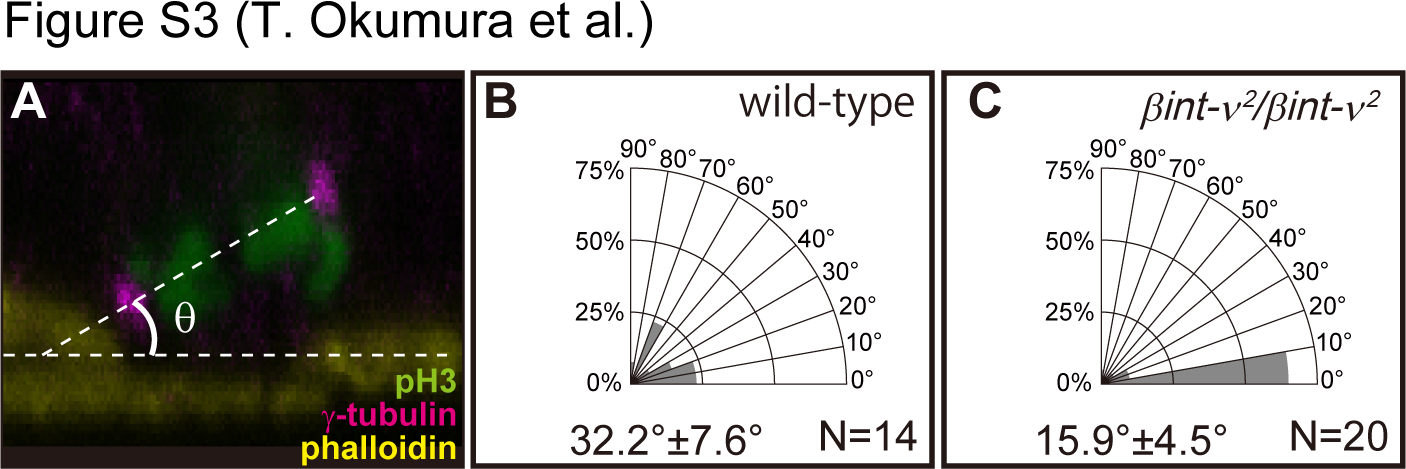

Supplement: Figure S3 — Angle between the spindle body and BM was frequently lower in mitotic ISCs of βint-ν mutants. (A) An example of mitotic ISCs stained with anti-pH3 antibody (green), anti-γ-tubulin antibody (magenta), and phalloidin (yellow). θ is an angle between the spindle body and BM indicated with broken lines. (B and C) The graphs showing frequencies of the angle (θ) measured in the PMG of wild-type (B) and βint-ν2 homozygous mutants (C). Values shown at each bottom are average ± S.E.M. (TIF) [file pone.0089387.s003.tif]

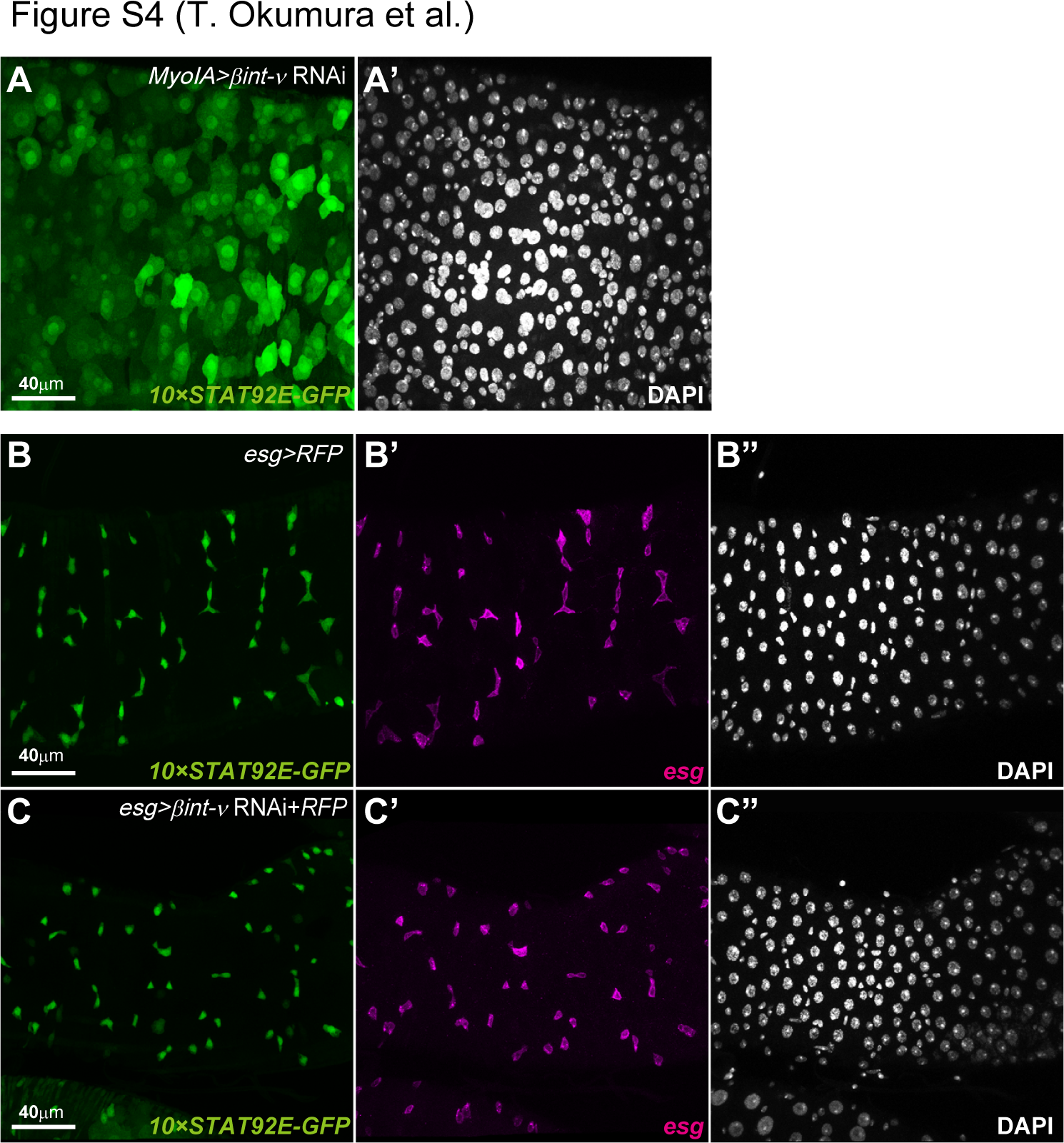

Supplement: Figure S4 — βint-ν RNAi in ECs but not in ISCs/EBs affected expression of 10×STAT92E-GFP . (A and A’) The PMG where βint-ν RNAi was performed with MyoIA-GAL4 driver. Abnormal expression of 10× STAT92E-GFP (green) was induced in the βint-ν RNAi midgut, compared with control (Figure 5A, C, and H). (B and C) The PMG where βint-ν RNAi was performed with esg-GAL4 driver. Expression of 10×STAT92E-GFP (green) was normal in both control (B) and βint-ν RNAi (C) flies. The esg-GAL4-driven expression was monitored with UAS-RFP (magenta in B’ and C’). Nuclei were stained with DAPI (white in B’’ and C’’). (TIF) [file pone.0089387.s004.tif]

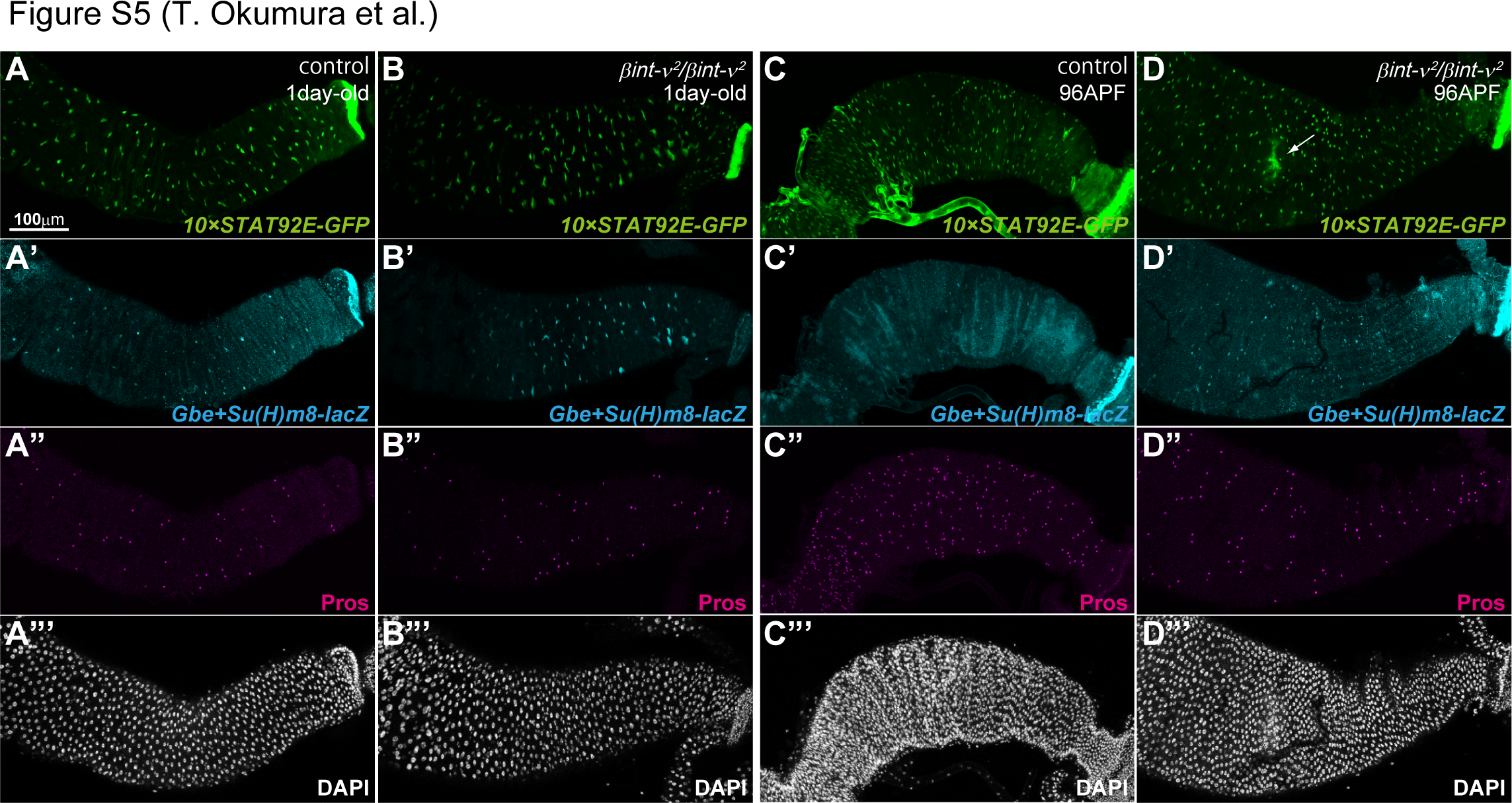

Supplement: Figure S5 — The midgut of βint-ν mutant did not show differentiation defects at the pupal stage and 1day-old adult stage. (A–D”’) The PMG of control and βint-ν 2 homozygotes at 1day-old adult (A–B) and 96 hour after puparium formation (APF) (C–D). Expression pattern of 10× STAT92E-GFP (green), Gbe-Su(H)m8-lacZ (cyan), and Pros (magenta) was not altered in βint-ν 2 midgut. Arrow in D indicates a signal from the yellow body in the gut lumen [74]). Nuclei were stained with DAPI (white). (TIF) [file pone.0089387.s005.tif]

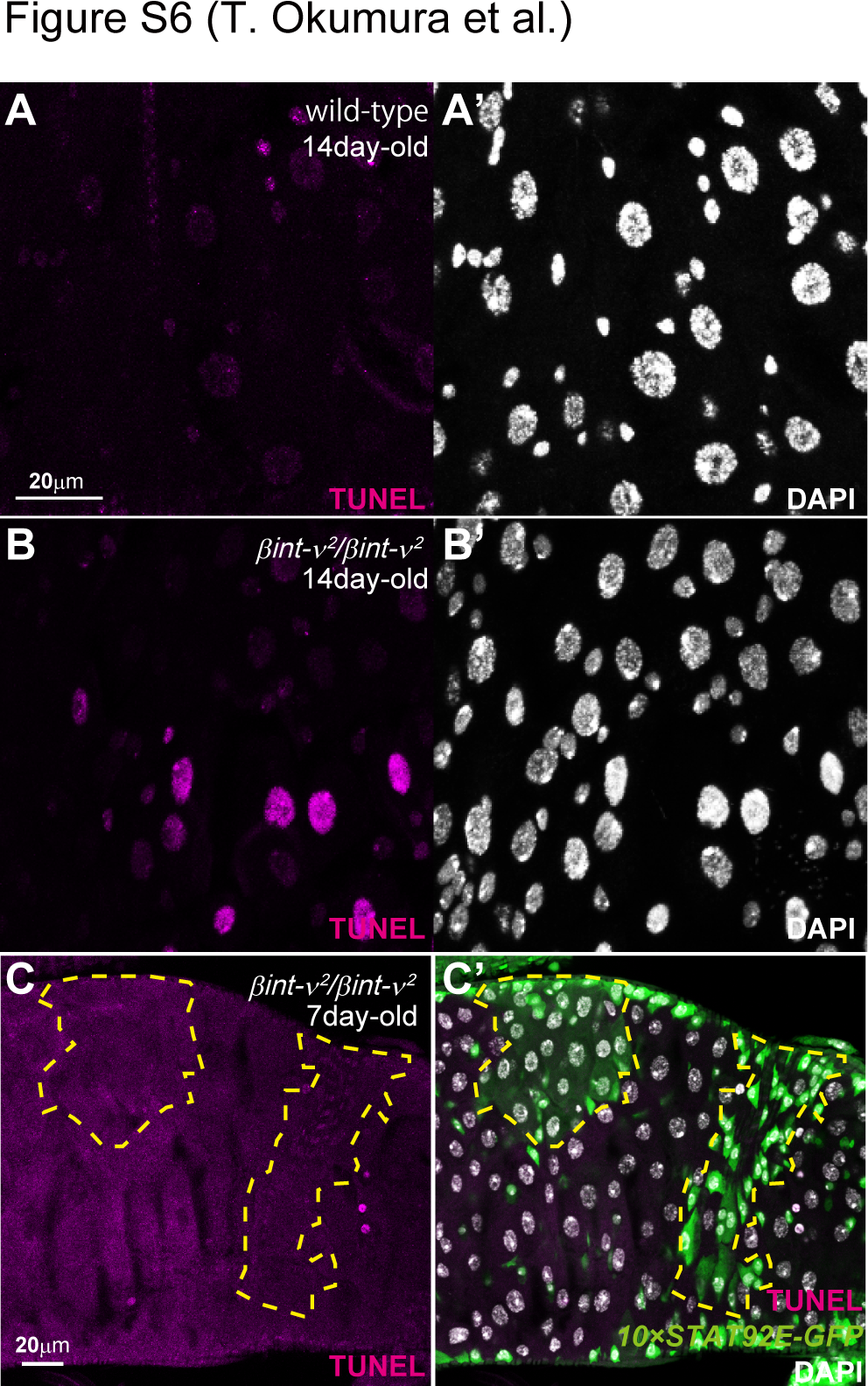

Supplement: Figure S6 — Faint increase of cell death in the regions with local defects caused in βint-ν mutant. (A and B) The PMG of wild-type and βint-ν 2 homozygotes at 14day-old, where TUNEL assay was performed. In wild-type (A), strong TUNEL signal (magenta) was frequently undetected, but, in the βint-ν 2 mutants (B), a slight increase of TUNEL-positive cells was observed at 14-days-old. (C) At 7-day-old flies, TUNEL-positive cells did not appear in the region of the local defects with ectopic JAK/STAT activation (indicated by broken lines) caused in βint-ν 2 midgut. Nuclei were stained with DAPI (white). (TIF) [file pone.0089387.s006.tif]

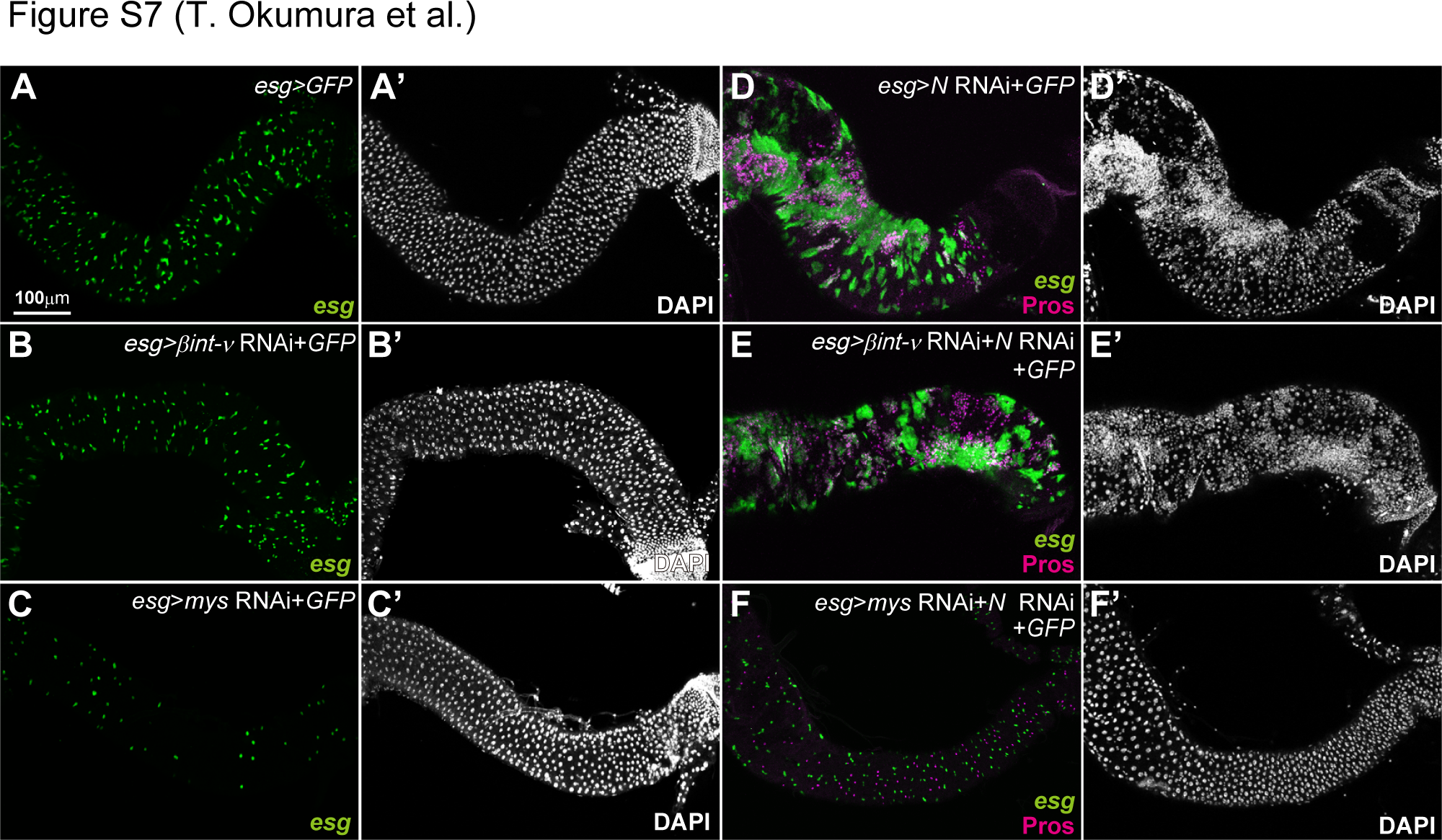

Supplement: Figure S7 — mys RNAi in ISCs/EBs affected maintenance of ISCs. (A–C) The PMG with non-RNAi (control)(A and A’), βint-ν RNAi (B and B’), and mys RNAi (C and C’) treatment using esg-GAL4 driver. In their treatments for 14 days, only mys RNAi caused a decrease in number of esg-positive cells (green). (D–F) The PMG with N RNAi (D and D’), βint-ν RNAi +N RNAi (E and E’), and mys RNAi +N RNAi (F and F’) treatments using esg-GAL4 driver. Growth of ISC-like (green) and ee-like (magenta) tumor induced by N RNAi was inhibited by mys RNAi but not by βint-ν RNAi. Nuclei were stained with DAPI (white). (TIF) [file pone.0089387.s007.tif]

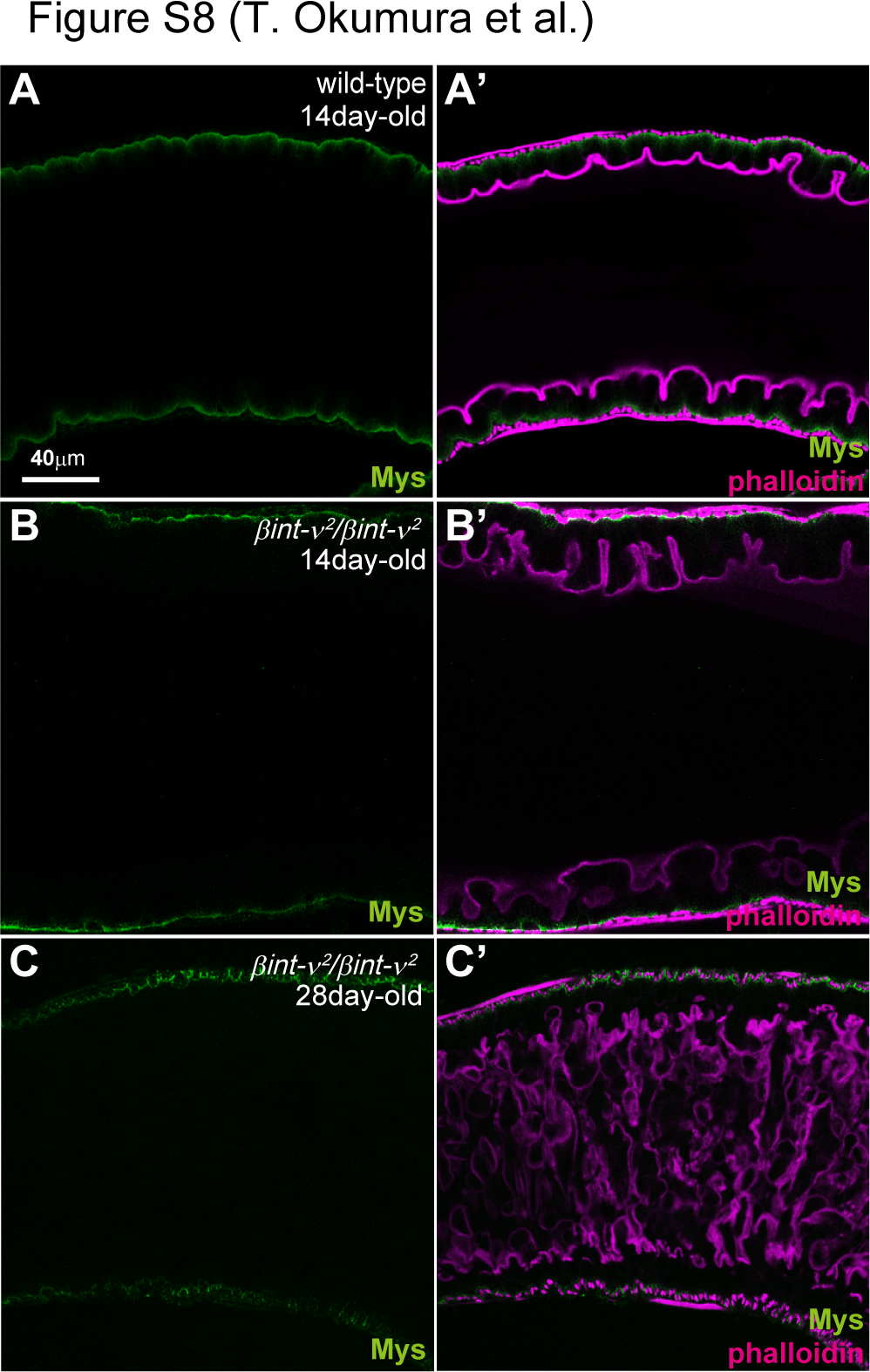

Supplement: Figure S8 — Distribution of Mys in the PMG was not affected by βint-ν mutation. (A–C) The PMG of wild-type at 14-day-old (A and A’), βint-ν 2 homozygote at 14-day-old (B and B’), and βint-ν 2 homozygote at 28-day-old (C and C’), stained with anti-Mys antibody (green) and phalloidin (magenta). Distribution pattern of Mys was not affected by βint-ν 2 homozygosity. (TIF) [file pone.0089387.s008.tif]
